# Circulating Blood-based Proteins in Psychopathology and Cognition: A Mendelian Randomization Study

**DOI:** 10.1001/jamapsychiatry.2025.0033

**Published:** 2025-05-01

**Authors:** Upasana Bhattacharyya, Jibin John, Max Lam, Jonah Fisher, Benjamin Sun, Denis Baird, Stephen Burgess, Chia-Yen Chen, Todd Lencz

**Affiliations:** 1 Institute of Behavioral Science, Feinstein Institutes for Medical Research, Manhasset, NY; 2 Division of Psychiatry Research, Zucker Hillside Hospital, Glen Oaks, NY; 3 Institute of Mental Health, Singapore; 4 Lee Kong Chian School of Medicine, Population and Global Health, Nanyang Technological University; 5 Biogen Inc., Cambridge, MA; 6 Harvard T.H. Chan School of Public Health, Cambridge, MA; 7 MRC Biostatistics Unit, University of Cambridge, Cambridge, UK; 8 Cardiovascular Epidemiology Unit, Department of Public Health and Primary Care, University of Cambridge, Cambridge, UK; 9 Departments of Psychiatry and Molecular Medicine, Zucker School of Medicine at Hofstra/Northwell, Hempstead, NY

## Abstract

**Importance:**

Peripheral (blood-based) biomarkers for psychiatric illness could benefit diagnosis and treatment, but research to date has typically been low-throughput, and traditional case-control studies are subject to potential confounds of treatment and other exposures. Large-scale 2-sample Mendelian Randomization (MR) can examine the potentially causal impact of circulating proteins on neuropsychiatric phenotypes without these confounds.

**Objective:**

To identify circulating proteins associated with risk for schizophrenia (SCZ), bipolar disorder (BIP), and major depressive disorder (MDD), as well as cognitive task performance (CTP).

**Design, Setting, and Participants:**

In a 2-sample MR design, significant proteomic quantitative trait loci (pQTLs) were used as candidate instruments, obtained from two large-scale plasma proteomics datasets: the UK Biobank Pharma Proteomics Project (UKB-PPP) (2,923 proteins/34,557 UK individuals) and deCODE Genetics (4,719 proteins / 35,559 Icelandic individuals).

**Main Outcome(s) and Measure(s):**

Outcome measures were summary statistics drawn from recent large-scale genome-wide association studies (GWAS) for SCZ (N_case_ = 67,323, N_control_ = 93,456), BIP (N_case_ = 40,463, N_control_ = 313,436), MDD (N_case_ = 166,773, N_control_ = 507,679), and CTP (N= 215,333). MR was carried out for each phenotype and proteins that showed statistically significant (Bonferroni-corrected *P* < 0.05) associations from MR analysis were used for pathway, protein-protein interaction (PPI), drug target enrichment and potential druggability analysis for each outcome phenotype separately.

**Results:**

MR analysis revealed 113 Bonferroni-corrected associations (46 novel) involving 91 proteins across the four outcome phenotypes. Immune-related proteins, such as interleukins and complement factors, showed pleiotropic effects across multiple outcome phenotypes. Drug target enrichment analysis provided support for repurposing of anti-inflammatory agents for SCZ, amantadine for BIP, retinoic acid for MDD, as well as duloxetine for CTP.

**Conclusions and Relevance:**

Identifying potentially causal effects of circulating proteins on neuropsychiatric phenotypes suggests potential biomarkers and offers insights for the development of innovative therapeutic strategies. The study also reveals pleiotropic effects of many proteins across different phenotypes, indicating shared etiology among serious psychiatric conditions and cognition.

## Introduction

Identification of biological factors underlying psychiatric conditions has been challenging, in part due to lack of direct access to central nervous system tissue. While studies of peripheral factors (e.g., circulating proteins) have the advantage of ease of collection, research in this area has typically been low-throughput and subject to potential confounds of treatment, hospitalization, and other environmental exposures common to psychiatric patients^1–3^.

Genomewide association studies (GWAS) represent a high-throughput, comprehensive, and unbiased tool for providing insights into psychopathology. Moreover, GWAS can provide in-depth mechanistic insights of disease pathogenesis by leveraging additional functional data^4–6^. Notably, the Mendelian Randomization (MR) approach^7^ permits the identification of putatively causal mechanisms, avoiding potentially confounding effects of environmental exposures while allowing for sample sizes orders of magnitude larger than conventional case-control studies.

Here, we provide the largest study to date utilizing MR to examine proteomic exposures as potential causal risk factors for psychiatric phenotypes. Importantly, the aim of our investigation is to provide evidence supporting or refuting putative exposures as putatively causal risk factors, not to estimate the absolute magnitude of specific proteomic effects; assumptions and potential confounds of MR are addressed in eAppendix 1. We employed blood-based proteomics data generated from two independent, large-scale cohorts (UK Biobank and deCODE Genetics) and well-powered GWAS for three psychiatric disorders: schizophrenia (SCZ), bipolar disorder (BIP), and major depressive disorder (MDD). Genetic architecture of these disorders is highly inter-correlated^8^, and all three disorders are phenotypically and/or genetically associated with deficits in cognitive task performance (CTP)^9–12^, which we included as an additional outcome phenotype. Downstream analyses were then conducted to yield pathophysiological insights and identify novel drug targets.

## Methods

### Overview

Candidate instruments for Mendelian Randomization were both cis-acting and trans-acting protein quantitative trait loci (pQTLs) derived from circulating plasma, drawn from two resources: the UK Biobank Pharma Proteomics Project (UKB-PPP) (2,923 proteins from 34,557 UK individuals) and deCODE Genetics (4,719 aptamers from 35,559 Icelandic individuals) (eTables 1a-1d). For outcomes, GWAS summary statistics were obtained for European-ancestry subjects from the most recent Psychiatric Genomics Consortium (PGC) reports for SCZ, BIP, and MDD, as well as the largest available non-overlapping dataset for CTP (detailed in the eMethods). Study participants for all GWAS utilized in the present study, including both exposure datasets (deCODE and UK Biobank) and all outcome datasets, provided written informed consent to protocols approved by local institutional review boards, as detailed in the original GWAS publications.

**Figure 1:**
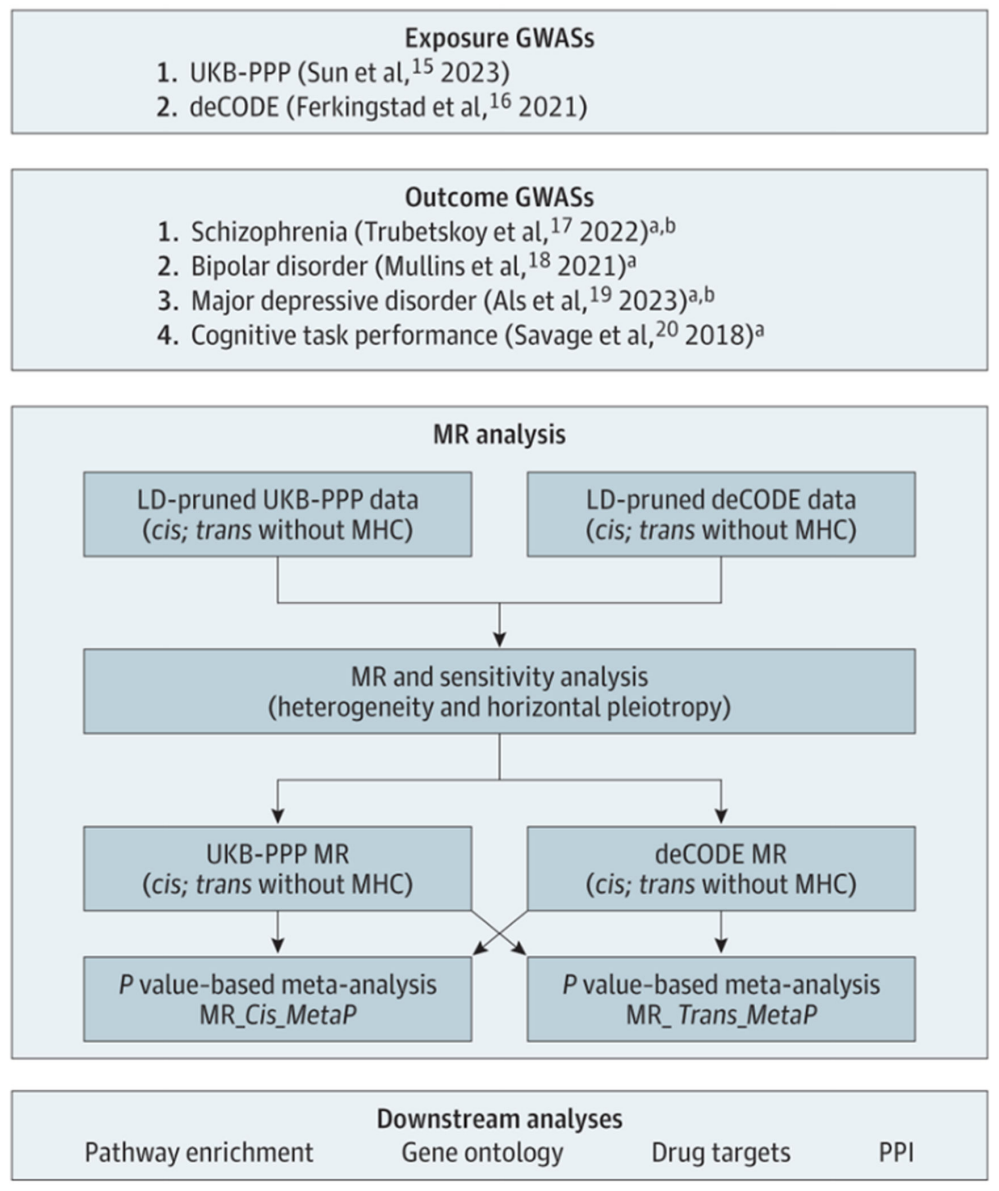
MR analysis workflow.

The IVW delta method from the ‘MendelianRandomization’ package^13,14^, was the primary approach to investigate the proteomic associations (separately for cis- and trans-pQTLS) for each phenotype (Figure 1, eFigure 1), followed by a series of sensitivity analyses (see eMethods) to ensure robustness of findings. Any significant associations (FDR<0.05) for which the MR Steiger test suggested reverse directionality (outcome>exposure) were tested for reverse causal directionality by using SCZ, BIP, MDD, or CTP as the exposure and UKBB-pQTL and/or deCODE-pQTL as outcome phenotypes.

To test potential confounding effects of substance use, MR analyses were performed after removing any SNPs significant in large-scale GWAS of tobacco and alcohol usage, represented as smoking days (CigDay; n=784,353) and drinking weeks (DrnkWk; n=2,965,643)^15^. To further test for potential pleiotropic effects of non-psychiatric diseases, we performed a phenomewide association study on all nominally significant (P<0.05) SNP instruments within all Bonferroni-significant genes using the IEU OpenGWAS database^16,17^, filtered using the keywords “cancer” and “disease.”

An extensive literature search was performed to determine potential novelty of significant MR results (eMethods). Finally, proteins that showed significant (Bonferroni-corrected *P*<0.05) effects from the cis-pQTL MR analysis were examined using pathway (EnrichR^18^), protein-protein interaction (PPI) (StringDb^19^), gene drug interaction(DGIdb 5.0^20–22^), druggability(DGIdb 5.0^20–22^), and drug target enrichment analyses (EnrichR^18^, DGIdb 5.0^20–22^) for each outcome phenotype separately. Detailed methods are reported in the supplemental online materials (eMethods).

## Results

### Cis association

**Figure 2:**
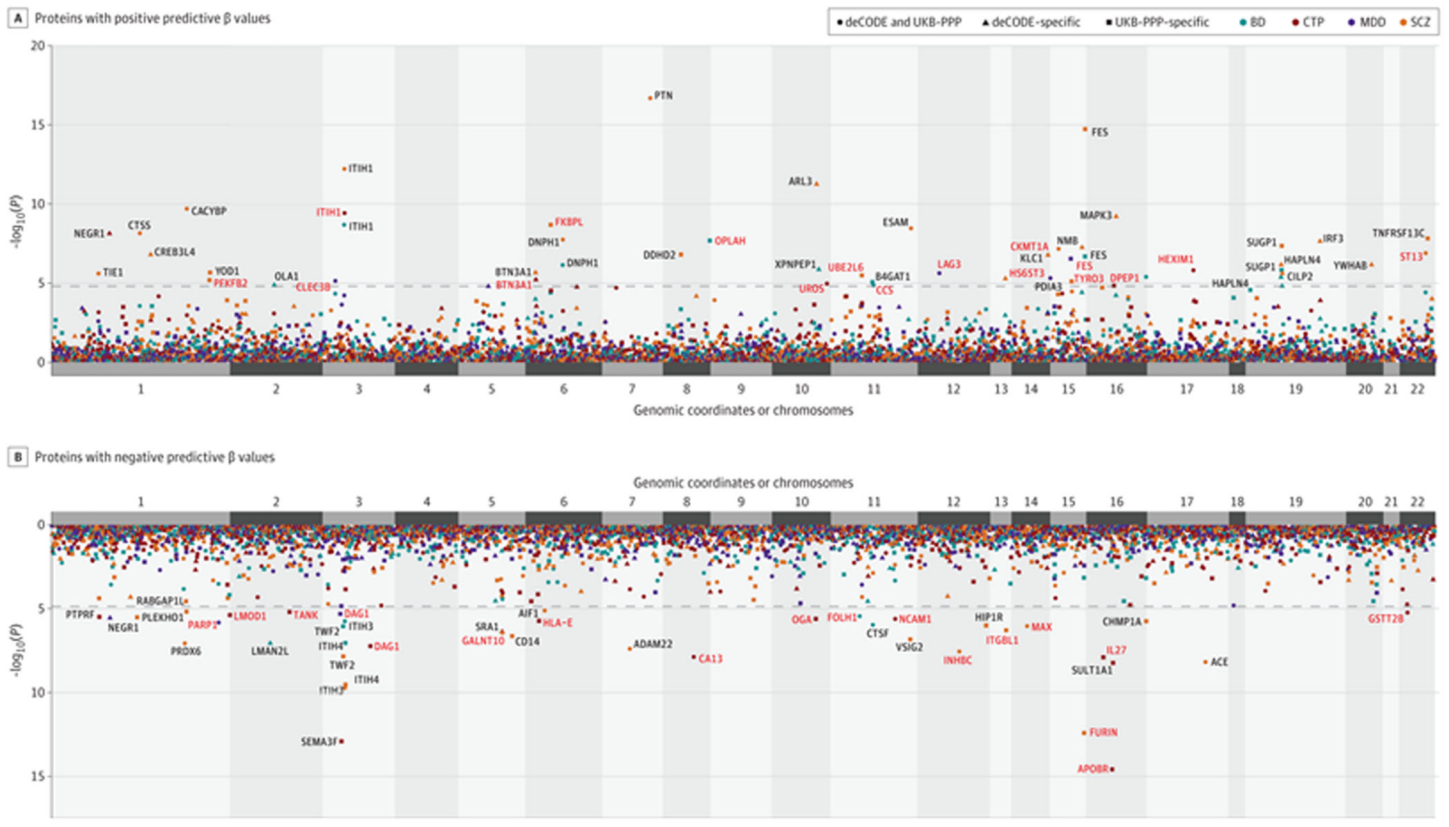
Manhattan plot showing findings from MR analysis for schizophrenia, bipolar disorder, major depressive disorder, and cognitive task performance, employing Cis- pQTLs from UKB-PPP and deCODE dataset as instrumental variables *Note*: Manhattan plots: (Top panel) proteins with positive predictive beta-values (Bottom panel) proteins with negative predictive beta-values relative to the traits investigated. X-axis: genomic coordinates/chromosomes; Y-axis: -log10p values of associations. Traits: Bipolar Disorder (turquoise), Cognitive ability (red), MDD (purple), Schizophrenia (orange). MR/Meta-Analysis: Meta-P (circle), deCODE: Results for deCODE specific MR analysis (triangle), UKB- PPP: Results for UKB-PPP specific MR analysis (square). Proteins based on more than one method, on multiple traits may be represented in the figure.

Mendelian Randomization analysis, employing cis-pQTLs as candidate instrumental variables, identified 77 proteins (*P*_BONF_<0.05) that may be causally associated with the susceptibility to psychiatric disorders and cognitive task performance (SCZ:47, BIP:17, MDD:7, CTP:19) (Table 1, Figure 2, eFigure 2A-D; all details in eTable 2a). In Figure 2, the direction of effect for CTP is reversed to maintain consistency with psychiatric conditions (i.e., poorer cognition / increased risk for psychiatric disorder). For example, Figure 2 shows that higher protein levels of ITIH1 are significantly associated with decreased cognitive performance and increased risk for SCZ and BIP. Using the MR Steiger test, all significantly associated proteins demonstrated the “correct” causal direction (i.e., the exposure is putatively causal to the outcome, rather than the reverse), however, 7 (SCZ:6, CTP:1) proteins were not statistically significant (*P*>0.05) for this test. We further tested these 7 proteins for reverse causality, performing MR in the opposite direction (SCZ or CTP as exposure); all 7 reverse analyses were significantly (*P*<0.05) “false” for causal directionality (eTable 2d). Moreover, no protein showed significant horizontal pleiotropy (all *P*>0.05 for the MR-Egger intercept test). Approximately 40% of findings employed a single instrumental SNP; seven of the remaining results (SCZ:3, CTP:3, BIP:1) demonstrated nominally significant (P<0.05) levels of heterogeneity (see forest plots, eFigures 3A-3BK). All findings remained significant (*P*<0.05) in MR-PRESSO, which is sensitive to weak instruments and pleiotropic outlier biases, in addition to the other sensitivity analyses such as weighted median, MRConMix, and MR IVW Robust. Only a scattering of results were not significant (*P*>0.05) in any of the other sensitivity analyses, and no proteins failed more than one sensitivity test. None of the IVs from our significant cis-pQTL findings were significant in CigDay. Only one IV from SUL1T1A1 (CTP) was significant in DrnkWk; after removing that IV, the protein no longer remained significant (eTable 2e). Only 7 significant proteins (SCZ: 4, BIP: 1, CTP: 2) contained SNP instruments that were significantly associated with non-psychiatric diseases in Phewas; none contained more than 1 such SNP, and disease associations ranged widely and did not suggest any clear confounding mechanism (eTable 2i). Additional results obtained when employing a less strict 5% false discovery rate threshold (FDR<0.05) are reported in the eResults (and eTable 2a).

An extensive literature search was conducted to identify any prior Transcriptome-Wide Association Studies (TWAS), Summary-data-based Mendelian Randomization (SMR), Mendelian Randomization (MR), or colocalization analyses in SCZ, BIP, MDD, and CTP (eTable 3a-c), using either blood-based or brain-based gene expression (eQTL) and/or proteomic (pQTL) data reference datasets. Our literature review revealed that 16 of the 47 Bonferroni-significant proteins for SCZ, 4/17 proteins for BIP, 6/7 for MDD, and 14/19 proteins for CTP had not been previously reported as significant (corrected for multiple comparisons) for that specific phenotype (Table 1, eTable 3b).

Our study comprises pQTL data from two cohorts, UKB-PPP and deCODE, which utilize different protein profiling platforms (Olink and SomaScan, respectively). Our MR results are consistent with a recent comprehensive study that reported a moderate level of correlation across these two distinct platforms^23^, particularly in the context of cis-pQTLs. Pearson correlation analysis revealed coefficients in the range of 0.46-0.53 for per-protein effect sizes obtained from MR analysis (eFigure 4A-D). Among the 77 proteins (*P*_BONF_<0.05) identified in our cis-pQTL MR analysis, 30 were assayed in both cohorts, and their associations with outcome phenotypes were replicated in both cohorts; 31 and 16 unique proteins were exclusively assayed in the UKB-PPP cohort and deCODE cohort respectively (eTable 2a).

### Trans association

**Figure 3:**
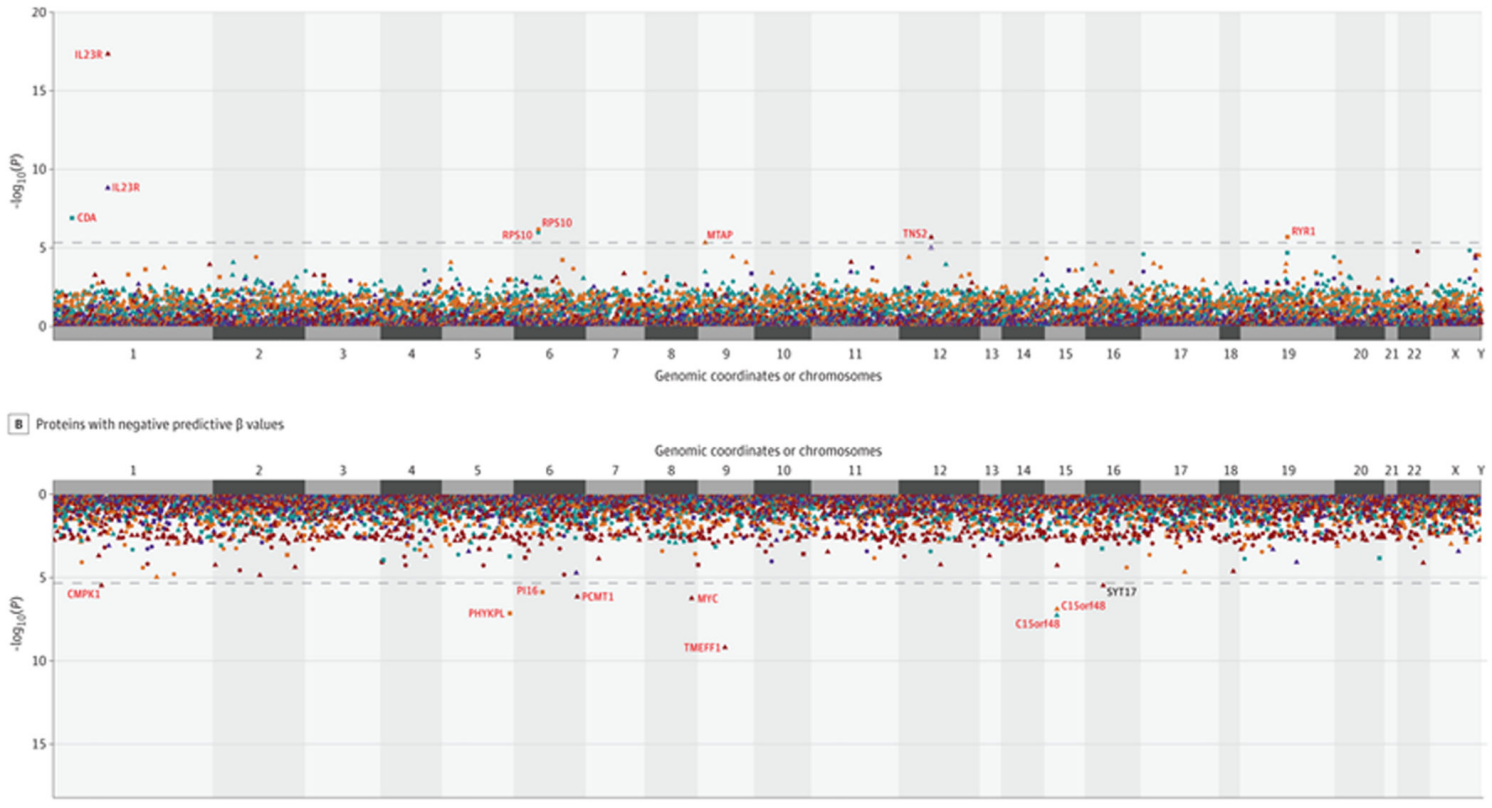
Manhattan plot showing findings from MR analysis for schizophrenia, bipolar disorder, major depressive disorder, and task performance employing Trans-pQTLs from UKB-PPP and deCODE datasets as instrumental variables *Note*: Manhattan plots: (Top panel) proteins with positive predictive beta-values (Bottom panel) proteins with negative predictive beta-values relative to the traits investigated. X-axis: genomic coordinates/chromosomes; Y-axis: -log10p values of associations. Traits: Bipolar Disorder (turquoise), Cognitive ability (red), MDD (purple), Schizophrenia (orange). MR/Meta-Analysis: Meta-P (circle), deCODE: Results for deCODE specific MR analysis (triangle), UKB- PPP: Results for UKB-PPP specific MR analysis (square). Proteins based on more than one method, on multiple traits may be represented in the figure.

MR analyses employing trans-pQTLs as candidate instrument variables identified 14 significant (*P*_BONF_<0.05) proteins (SCZ:6, BIP:3, MDD:1, CTP:7) that may be causally associated with susceptibility to psychiatric disorders and cognitive performance (Table 1, eTable 2b, Figure 3; eFigure 2A-D). Our systematic review indicated that, except for SYT17 (CTP), none of these findings have been previously reported, even at nominal levels of statistical significance (Table 1, eTable 3a-c). All significant proteins in the trans-pQTL analyses demonstrated correct directionality, and all except for one (RYR1) were significant (*P*<0.05) on the MR Steiger test. In the reverse causality analysis, RYR1 was strongly significant, suggesting a potentially causal effect of schizophrenia on RYR1 levels (eTable 2d). Only PI16 (SCZ) exhibited significant horizontal pleiotropy, and 4 proteins (SCZ:2, CTP:2) exhibited nominal significance (*P*<0.05) for heterogeneity (see forest plots in eFigures 3A-BK). Unlike for cis-pQTLs, nearly all trans-pQTL findings came from MR analysis using more than one instrumental variable (eTable 2). Furthermore, all findings remained significant (*P*<0.05) using more robust MR methods such as MR-PRESSO, MR-IVW robust, weighted median, weighted mode, MR Egger Robust, MRcML, and MRConMix (eTable 2b). None of the IVs from our trans-pQTL findings were significant in CigDay. Only one IV from PI16 (SCZ) was significant in DrnkWk. After removing that IV, the protein remained significant in MR (eTable 2e). Only 2 significant proteins (BIP: 1, CTP: 1) contained a single SNP instrument significantly associated with non-psychiatric diseases in Phewas (eTable 2i). Additional results obtained when employing a less strict 5% false discovery rate threshold (FDR<0.05) are reported in the eResults (and eTable 2b).

Unlike for cis-pQTLs, we observed only modest correlations of MR results across the two proteomics platforms, with coefficients across all four phenotypes in the range of 0.08 to 0.15 for per-protein effect sizes (eFigure 5A-D), which is consistent with an earlier report, mentioned previously^24^. Only 1 protein (RPS10) among the 14 unique proteins (*P*_BONF_<0.05) identified in our study was assayed by both deCODE and UKB-PPP, and its associations with SCZ and BIP were replicated in both platforms.

### Overlap across phenotypes

**Figure 4:**
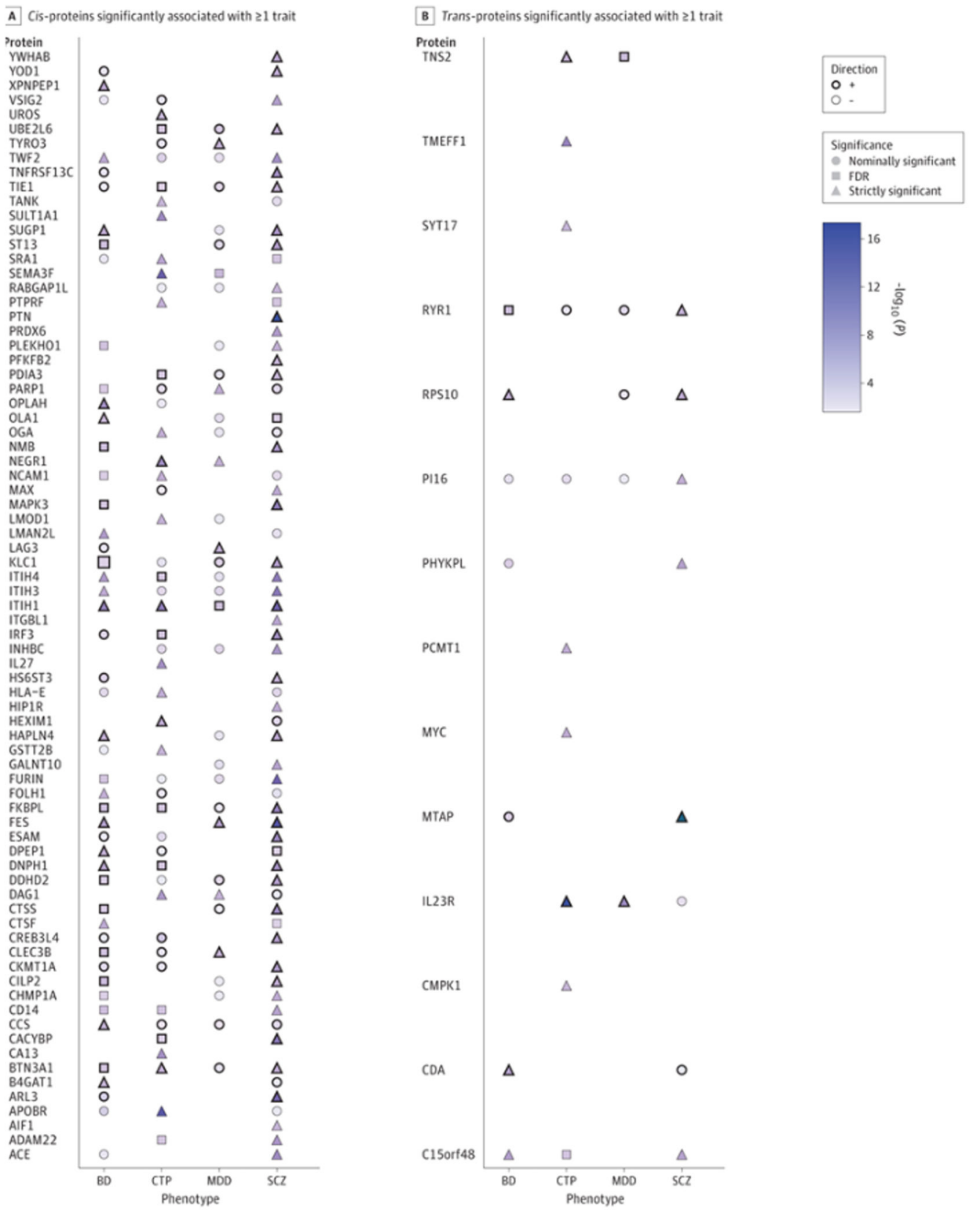
Cis-pQTL MR results across traits A. Venn Diagram showing shared significant (P-Bonf<0.05) cis-protein across four phenotypes B. Cis-Proteins significantly associated with at least one trait are presented in the figure. Note: The strength of MR association p-values is represented by a blue gradient across data values. The strength of association is further annotated for easy reference, nominally significant p < 0.05 (circle), FDR significant P_scz_ < 1.58 x 10^-3^ P_BIP_ < 7.3 x 10^-4^, P_MDD_ < 4.9 x 10^-3^, P_CTP_ < 1 x 10^-3^ (square), and Bonferroni significant p < 1.31x 10^-5^ (triangle) are annotated accordingly. C. Venn Diagram showing shared significant (P-Bonf<0.05) trans-protein across four phenotypes D. Trans-Proteins significantly associated with at least one trait are presented in the figure. *Note:* Proteins significantly associated with at least one trait are presented in the figure. The strength of MR association p-values is represented by a blue gradient across data values. The strength of association is further annotated for easy reference, nominally significant p < 0.05 (circle), FDR significant P_scz_ < 1.8 x 10-4, P_BIP_ < 3.81 x 10^-5^, P_MDD_ < 1.90 x 10^-5^, P_CTP_ < 2 x 10-4 (square) and Bonferroni significant p < 7.3 x 10^-6^(triangle) are annotated accordingly.

We observed considerable evidence for pleiotropic effects of many proteins across phenotypes (Figures 4A and 4C). In Figure 4B, ITIH4 demonstrates significant associations with SCZ and BIP, FDR-level association with CTP, and nominally significant association with MDD. In parallel, TWF2 emerged as another protein with involvement in all four phenotypes (Figures 4B, 4D). Interestingly, IL23R, a novel discovery in trans-pQTL analyses for MDD, displays an inverse relationship between cognition and depression (Figure 4D). PARP1 also displays pleiotropic relationships in opposite directions for schizophrenia relative to the other disorders; otherwise, all observed pleiotropic relationships are concordant for up- or down-regulation.

### Pathway enrichment

Pathway enrichment analyses of Bonferroni-corrected cis-pQTL results revealed that SCZ, BIP, MDD and CTP associated proteins are enriched in immune response pathways (eTable 4a). SCZ-associated proteins were also enriched for proteins involved in neurodegenerative disorders such as Alzheimer’s and Parkinson’s. BIP-associated proteins were significantly enriched in platelet-related and peptidase-mediated proteolysis pathways. MDD-associated proteins were enriched in neurodevelopmental and immune function pathways, as well as signalling pathways related to the MAPK cascade, which is involved in neuronal plasticity, function, and survival^25^. CTP-assocated proteins were enriched for neurodevelopmental and synaptic pathways such as Axon Guidance and Axonogenesis.

### Protein-Protein Interaction

Bonferroni-significant cis-pQTL proteins associated with SCZ and BIP demonstrated significant PPI enrichment (observed:expected edges for SCZ=28:11, *P*=1.19E-05; BIP=4:0, *P*=0.00139). MAPK3, FURIN, ITIH1, ITIH3 and ITIH4 were found to constitute the main hub of the PPI network in SCZ (eFigure 6A). A previous study of schizophrenia-associated CNV genes also identified MAPK3 as the most prominent network hub in PPI networks within the 16p11.2 proximal region^26^. Additionally, TIE1 and ESAM were found to constitute a separate network, potentially related to blood-brain barrier function. ITIH1 was an important node in bipolar disorder (eFigure 6B). The PPI networks for other phenotypes did not reveal statistically significant enrichment (*P*>0.05). (eFigure 6C, 6D).

### Drug Target Enrichment

We evaluated potential druggability of the Bonferroni-corrected, cis-associated proteins in multiple ways, as elaborated in the eMethods. Using EnrichR^18^, we identified several of the associated proteins (including PDIA3, ITIH4, ITIH3, CD14, AIF1, and CTSS) are targets of established nonsteroidal anti-inflammatory drugs such as rofecoxib and celecoxib (Table 1, eTable 4b). In addition to known psychiatric agents such as modafinil and valproic acid, SCZ-associated proteins were found to be enriched for targets of calcium channel blockers and nicotine. For BIP, in addition to known antipsychotic and mood-stabilizing drugs such as olanzapine, carbamazepine, and valproic acid, associated proteins were enriched for targets of amantadine, which has anti-NMDA activity and has been suggested as a potential therapeutic option in bipolar disorder^27^. Among the proteins associated with MDD, NEGR1 and TYRO3 were found to be targets of a selective serotonin reuptake inhibitor (paroxetine) and an MAO inhibitor (phenelzine). Additionally, PARP, NEGR1, FES, TYRO3, and DAG1 were targets of retinoic acid. Proteins associated with cognition were enriched for targets of yohimbine, which has been studied for nootropic and anxiety-reducing purposes^27^. Utilizing DGIdb5.0^22^, we identified 13 actionable drug targets for SCZ, 7 for BIP, 5 for MDD, and 9 for CTP (P_BONF_<0.05; Table 1), including FES, MAPK3, ACE, CD14, ITIH3, HLA-E, and NCAM16 (eTable 5a). Several of these proteins are targets of drugs already in use for psychiatric indications, such as ITIH3 with clozapine and ACE with sertraline. Additionally, a subset of these targets may identify potential opportunities for drug repurposing, including methotrexate (an immune-suppressant and antimetabolite) for bipolar disorder and duloxetine (an antidepressant) for cognitive task performance. Perhaps most importantly, almost all proteins identified in our study, with a few exceptions, were indicated to be druggable (eTable 5b).

## Discussion

The present study identified evidence of 113 Bonferroni-corrected putatively causal associations between 91 proteins and one or more of our target phenotypes. Compared to the relevant existing literature across both blood- and brain-based studies of protein levels and/or gene expression, approximately half of these associations are significant for the first time in our study, including numerous pQTLs identified *in trans*. Loosening the statistical threshold to the FDR<0.05 level resulted in several hundred associations with widespread pleiotropic effects detected (Figure 4; eTable 2). Notably, two-sample Mendelian Randomization relies on a set of assumptions about the nature of the datasets and instruments utilized^28^, and results were robust to an extensive series of sensitivity analyses designed to control for violations of those assumptions^29^. For example, all our findings remain significant in both the MR-PRESSO and MR-IVW Robust methods, which are robust to heterogeneity/outliers amongst genetic variants. Additionally, nearly all findings remained significant using MR-Con-Mix, which is not only robust to outliers but also sensitive to variance parameters and the inclusion/exclusion of genetic variants. Furthermore, nearly all Bonferroni-significant proteins retained significance in MR-cML, a method robust to violations of all three instrumental variable assumptions.

It is noteworthy that many of the proteins identified in our study are related to immune/inflammatory function, including interleukins, toll-like receptors, and complement factors. While prior case-control studies^30^ have identified heightened levels of pro-inflammatory cytokines such as IL-6, IL-8, and IL-1β in patients with serious mental illness, it is noteworthy that none of these emerged as significant in our analysis, consistent with evidence that medication^31,32^ and other state-related factors influence the levels of these biomarkers in patients with serious mental illness. Similarly, prior case-control research has replicably observed elevated blood levels of CRP in individuals with schizophrenia; but our results indicated a *reduced* level of circulating CRP was associated with genetic risk for schizophrenia, suggesting that previous findings may be state-related^33,34^. Moreover, the strongest individual signal in the present study was IL23R; higher levels of IL23R were associated with increased risk of depression and poorer cognition. While elevated IL-23 has been associated with depression in patients with psoriatic arthritis^35^ (for which IL-23 is a major causal factor)^36^, there have been mixed reports concerning the correlation between IL-23 levels in the blood of patients with idiopathic depression^37,38^.

More broadly, blood-based inflammatory markers identified in the present study should be tested as potential early indicators of the risk of developing psychiatric disorders. It is worth noting that most prior case-control studies have utilized low-throughput methods to assess circulating proteins; only a few very recent studies have utilized the Olink or SomaScan platform to assess a large panel of proteins in serious mental illness^39–43^. Although these studies employed relatively small sample sizes and did not converge on specific individual proteins, their results suggest that inflammatory proteins and growth factors may be fruitful avenues for further study. Studies in patients at clinical high risk for psychosis, or in the first episode of illness, may be particularly informative due to relative lack of cumulative medication exposure, as well as providing data on the potential prognostic value of such blood-based proteomic biomarkers^44^.

In addition to immune and inflammatory proteins, our study was able to implicate many new proteins/genes by leveraging the large sample sizes available in our proteomic reference panels (eTable 3). For example, the apolipoprotein B receptor (APOBR) was the strongest cis-pQTL signal associated with cognitive task performance in the present study, and has not been previously reported in comparable studies. Circulating levels of ApoB have been associated with risk for Alzheimer’s disease^45^ and age-related cognitive decline^46,47^. Similarly, increased levels of circulating PCMT1, which has neuroprotective functions in animal models of cognitive aging^48^, were associated with better cognitive task performance. We also report, for the first time, a putatively causal relationship between reduced levels of folate hydrolase I (FOLH1) and increased risk for bipolar disorder (as well as increased schizophrenia risk at nominal *P*<0.05). In the brain, FOLH1 catalyzes the breakdown of N-acetylaspartylglutamate into glutamate, thereby serving as a high-level control of neurotransmission and excitotoxicity^49^; reductions in the levels of FOLH1 have been reported in post-mortem brain tissue of patients with schizophrenia^50^. Additionally, reduced levels of syntaxin binding protein 1 (STXBP1, critical to release of neurotransmitters from synaptic vesicles) were associated with schizophrenia; loss of function mutations in this gene cause a range of neurodevelopmental disorders^51^.

Intriguingly, the pathway enrichment analysis in schizophrenia also reveals shared proteins with neurodegenerative disorders such as Alzheimer’s and Parkinson’s Disease, consistent with a recent brain-based proteomic study^52^ demonstrating unexpected overlap between schizophrenia and neurologic disorders. This raises the possibility of immune protein-mediated neurodegeneration as a potential contributing factor to the risk of schizophrenia. Pathway enrichment analysis for bipolar disorder found enrichment of peptidase-mediated proteolysis pathways. Regulated proteolysis is a fundamental process for neuronal maintainence^53^ and synaptic plasticity^54^.

A primary goal of GWAS is the identification of novel drug targets^53^, yet this process is complicated by the lack of specificity of the broad genomic loci typically identified by GWAS. Omics studies, such as the present study’s examination of pQTLs, permit the conversion of GWAS signals into specific drug targets^55^. Our drug target enrichment analyses successfully identified already-approved medications for each of the three psychiatric disease phenotypes (eTable 4b), providing a positive control for our approach.

## Limitations

The present study attempted to understand the pathophysiology of brain-related phenotypes with proteomic measures derived from blood, rather than brain. Consequently, it is likely that we were unable to detect effects of central nervous system proteins that have limited expression in blood, although several of our novel findings are proteins primarily expressed in brain. The exposures (protein levels) were measured in both the UK Biobank and deCODE cohorts at a single timepoint in adulthood and may not capture fluctuating effects of protein levels across the lifespan. However, our results should provide a reliable guide as to the direction of effect of the exposure on the outcome, to the extent that the direction of effect of the genetic variants on the exposure, and the exposure on the outcome, are consistent over time. Our results may be vulnerable to selection bias, as UK Biobank participants are not representative of the general UK population^56^, and differential selection patterns can lead to spurious genetic associations. However, simulations have shown that moderate selection bias does not lead to substantial Type 1 error rates in realistic scenarios^57^. Moreover, there was significant correspondence between results derived from UK Biobank vs. deCODE, which had a substantially different recruitment process. Finally, Mendelian Randomization represents an indirect statistical test of association; future studies will be needed to directly assess the identified proteins in patients diagnosed with psychiatric disorders.

## Conclusions

While the present study and the prior case-control literature point to a role for peripheral inflammatory proteins in psychiatric conditions, our results suggest novel specific inflammatory proteins. Results provide support for repurposing of anti-inflammatory agents for SCZ, amantadine for BIP, retinoic acid for MDD, and duloxetine for CTP. Future work, including longitudinal studies in patient and high-risk cohorts, is required to determine if these results might suggest potential blood-based biomarkers for neuropsychiatric phenotypes. Since Mendelian Randomization is designed to nominate putatively causal and potentially modifiable risk factors, identifying individuals with abnormal levels of these proteins may be worthwhile.

## Supplementary Material

Supplementary material

## Figures and Tables

**Table 1:** Summary of Results The following table summarizes the number of significant proteins identified for each of the four outcome phenotypes. While we have emphasized the highest confidence (Bonferroni-significant) results in the text above, we also present results at the FDR level of significance in the Table below, as well as eTables 4c, 4d, 5c, and 5d.

Phenotypes	cis-Pqtl MR results	cis-pQTL Novel	Gene sets (# sets)	Drug Targets Enrichment (# drugs)	Drug Targets (DGIdb)	Druggable (DGIdb)	trans-pQTL MR results	trans-pQTL novel
SCZ	47 (129)	10 (54)	6 (131)	182 (64)	13 (54)	39 (103)	6 (21)	7 (20)
BIP	17 (55)	4 (21)	26 (27)	338 (624)	7 (24)	14 (44)	3 (6)	3 (7)
MDD	7 (15)	6 (13)	16 (46)	49 (35)	5 (7)	7 (14)	1 (3)	1 (3)
CTP	19 (74)	14 (52)	3 (46)	5 (14)	9 (30)	16 (57)	6 (31)	6 (31)

*Note*: Numbers in Table represent number of significant results at Bonferroni significance (number of results at FDR<0.05 in parentheses).

## Key Points

### Question

Are there directional associations between blood levels of specific proteins and either risk for serious psychiatric disorders or cognitive task performance?

### Findings

In this 2-sample Mendelian Randomization study, circulating levels of 91 proteins were significantly associated with schizophrenia, bipolar disorder, major depressive disorder, and/or cognitive task performance. These include a number of immune-related proteins such as interleukins and complement factors.

### Meaning

These proteins, which may be causally related to psychiatric and cognitive phenotypes, suggest novel treatment targets and could lead to blood-based biomarkers unconfounded by state-related factors.

## References

[1] Harsanyi S, Kupcova I, Danisovic L, Klein M (2022). Selected biomarkers of depression: What are the effects of cytokines and inflammation?. Int J Mol Sci.

[2] Upthegrove R, Khandaker GM (2020). Cytokines, oxidative stress and cellular markers of inflammation in schizophrenia. Curr Top Behav Neurosci.

[3] Vega-Núñez A, Gómez-Sánchez-Lafuente C, Mayoral-Cleries F (2022). Clinical value of inflammatory and neurotrophic biomarkers in bipolar disorder: A systematic review and meta-analysis. Biomedicines.

[4] Yang C, Fagan AM, Perrin RJ, Rhinn H, Harari O, Cruchaga C (2022). Mendelian randomization and genetic colocalization infer the effects of the multi-tissue proteome on 211 complex disease-related phenotypes. Genome Med.

[5] Gu X, Dou M, Su W (2022). Identifying novel proteins underlying schizophrenia via integrating pQTLs of the plasma, CSF, and brain with GWAS summary data. BMC Med.

[6] Liu J, Cheng Y, Li M, Zhang Z, Li T, Luo XJ (2023). Genome-wide Mendelian randomization identifies actionable novel drug targets for psychiatric disorders. Neuropsychopharmacology.

[7] Smith GD, Ebrahim S (2004). Mendelian randomization: prospects, potentials, and limitations. Int J Epidemiol.

[8] Lee SH, Ripke S, Cross-Disorder Group of the Psychiatric Genomics Consortium (2013). Genetic relationship between five psychiatric disorders estimated from genome-wide SNPs. Nat Genet.

[9] Van Rheenen TE, Lewandowski KE, Lipschitz JM, Burdick KE (2019). Conducting clinical studies targeting cognition in psychiatry: guiding principles and design. CNS Spectr.

[10] Lam M, Trampush JW, Yu J (2017). Large-scale cognitive GWAS meta-analysis reveals tissue-specific neural expression and potential nootropic drug targets. Cell Rep.

[11] Lam M, Chen CY, Hill WD (2022). Collective genomic segments with differential pleiotropic patterns between cognitive dimensions and psychopathology. Nat Commun.

[12] Lam M, Chen CY, Ge T (2021). Identifying nootropic drug targets via large-scale cognitive GWAS and transcriptomics. Neuropsychopharmacology.

[13] Yavorska OO, Burgess S (2017). MendelianRandomization: an R package for performing Mendelian randomization analyses using summarized data. Int J Epidemiol.

[14] Burgess S, Davey Smith G, Davies NM (2019). Guidelines for performing Mendelian randomization investigations: update for summer 2023. Wellcome Open Res.

[15] Saunders GRB, Wang X, Chen F (2022). Genetic diversity fuels gene discovery for tobacco and alcohol use. Nature.

[16] Hemani G, Zheng J, Elsworth B (2018). The MR-Base platform supports systematic causal inference across the human phenome. Elife.

[17] Elsworth B, Lyon M, Alexander T The MRC IEU OpenGWAS data infrastructure. bioRxiv.

[18] Kuleshov MV, Jones MR, Rouillard AD (2016). Enrichr: a comprehensive gene set enrichment analysis web server 2016 update. Nucleic Acids Res.

[19] Szklarczyk D, Kirsch R, Koutrouli M (2023). The STRING database in 2023: protein-protein association networks and functional enrichment analyses for any sequenced genome of interest. Nucleic Acids Res.

[20] Freshour SL, Kiwala S, Cotto KC (2021). Integration of the Drug-Gene Interaction Database (DGIdb 4.0) with open crowdsource efforts. Nucleic Acids Res.

[21] Li X, Jiang L, Xue C, Li MJ, Li M (2022). A conditional gene-based association framework integrating isoform-level eQTL data reveals new susceptibility genes for schizophrenia. Elife.

[22] Cannon M, Stevenson J, Stahl K (2024). DGIdb 5.0: rebuilding the drug-gene interaction database for precision medicine and drug discovery platforms. Nucleic Acids Res.

[23] Eldjarn GH, Ferkingstad E, Lund SH (2023). Large-scale plasma proteomics comparisons through genetics and disease associations. Nature.

[24] Krzystanek M, Pałasz A (2020). Possibility of a new indication for amantadine in the treatment of bipolar depression-case series study. Pharmaceuticals (Basel).

[25] Pérez-Sen R, Queipo MJ, Gil-Redondo JC (2019). Dual-Specificity Phosphatase Regulation in Neurons and Glial Cells. Int J Mol Sci.

[26] Blizinsky KD, Diaz-Castro B, Forrest MP (2016). Reversal of dendritic phenotypes in 16p11.2 microduplication mouse model neurons by pharmacological targeting of a network hub. Proc Natl Acad Sci U S A.

[27] Singewald N, Schmuckermair C, Whittle N, Holmes A, Ressler KJ (2015). Pharmacology of cognitive enhancers for exposure-based therapy of fear, anxiety and trauma-related disorders. Pharmacol Ther.

[28] Sanderson E, Glymour MM, Holmes MV (2022). Mendelian randomization. Nat Rev Methods Primers.

[29] Slob EAW, Burgess S (2020). A comparison of robust Mendelian randomization methods using summary data. Genet Epidemiol.

[30] Zhang Y, Wang J, Ye Y (2023). Peripheral cytokine levels across psychiatric disorders: A systematic review and network meta-analysis. Prog Neuropsychopharmacol Biol Psychiatry.

[31] Martins PLB, Moura IA, Mendes G (2023). Immunoinflammatory and oxidative alterations in subjects with schizophrenia under clozapine: A meta-analysis. Eur Neuropsychopharmacol.

[32] Ramos-Méndez MA, Tovilla-Zárate CA, Juárez-Rojop IE (2023). Effect of risperidone on serum IL-6 levels in individuals with schizophrenia: a systematic review and meta-analysis. Int J Psychiatry Clin Pract.

[33] Lestra V, Romeo B, Martelli C, Benyamina A, Hamdani N (2022). Could CRP be a differential biomarker of illness stages in schizophrenia? A systematic review and meta-analysis. Schizophr Res.

[34] Orsolini L, Sarchione F, Vellante F (2018). Protein-C Reactive as Biomarker Predictor of Schizophrenia Phases of Illness? A Systematic Review. Curr Neuropharmacol.

[35] Tabra SA, Abd Elghany SE, Amer RA, Fouda MH, Abu-Zaid MH (2022). Serum interleukin-23 levels: relation to depression, anxiety, and disease activity in psoriatic arthritis patients. Clin Rheumatol.

[36] Lee BW, Moon SJ (2023). Inflammatory Cytokines in Psoriatic Arthritis: Understanding Pathogenesis and Implications for Treatment. Int J Mol Sci.

[37] Gałecka M, Bliźniewska-Kowalska K, Orzechowska A (2021). Inflammatory versus Anti-inflammatory Profiles in Major Depressive Disorders-The Role of IL-17, IL-21, IL-23, IL-35 and Foxp3. J Pers Med.

[38] Kim JW, Kim YK, Hwang JA (2013). Plasma Levels of IL-23 and IL-17 before and after Antidepressant Treatment in Patients with Major Depressive Disorder. Psychiatry Investig.

[39] Eren F, Schwieler L, Orhan F (2023). Immunological protein profiling of first-episode psychosis patients identifies CSF and blood biomarkers correlating with disease severity. Brain Behav Immun.

[40] Isgren A, Göteson A, Holmén-Larsson J (2022). Cerebrospinal fluid proteomic study of two bipolar disorder cohorts. Mol Psychiatry.

[41] Idemoto K, Ishima T, Niitsu T (2021). Platelet-derived growth factor BB: A potential diagnostic blood biomarker for differentiating bipolar disorder from major depressive disorder. J Psychiatr Res.

[42] Korhonen L, Paul ER, Wåhlén K (2023). Multivariate analyses of immune markers reveal increases in plasma EN-RAGE in first-episode psychosis patients. Transl Psychiatry.

[43] Xu F, Su Y, Wang X, Zhang T, Xie T, Wang Y (2024). Olink proteomics analysis uncovers inflammatory proteins in patients with different states of bipolar disorder. Int Immunopharmacol.

[44] Guan F, Ni T, Zhu W (2022). Integrative omics of schizophrenia: from genetic determinants to clinical classification and risk prediction. Mol Psychiatry.

[45] Araújo DC, Veloso AA, Gomes KB (2022). A Novel Panel of Plasma Proteins Predicts Progression in Prodromal Alzheimer’s Disease. J Alzheimers Dis.

[46] Lutski M, Weinstein G, Goldbourt U, Tanne D (2019). Plasma Lipids, Apolipoproteins, and Subsequent Cognitive Decline in Men with Coronary Heart Disease. J Alzheimers Dis.

[47] Pokharel Y, Mouhanna F, Nambi V (2019). ApoB, small-dense LDL-C, Lp(a), LpPLA2 activity, and cognitive change. Neurology.

[48] Qin Z, Dimitrijevic A, Aswad DW (2015). Accelerated protein damage in brains of PIMT+/- mice; a possible model for the variability of cognitive decline in human aging. Neurobiol Aging.

[49] Barinka C, Sácha P, Sklenár J (2004). Identification of the N-glycosylation sites on glutamate carboxypeptidase II necessary for proteolytic activity. Protein Sci.

[50] Tsai G, Passani LA, Slusher BS (1995). Abnormal excitatory neurotransmitter metabolism in schizophrenic brains. Arch Gen Psychiatry.

[51] Xian J, Parthasarathy S, Ruggiero SM (2022). Assessing the landscape of STXBP1-related disorders in 534 individuals. Brain.

[52] Wingo AP, Liu Y, Gerasimov ES (2023). Sex differences in brain protein expression and disease. Nat Med.

[53] Ciechanover A, Kwon YT (2015). Degradation of misfolded proteins in neurodegenerative diseases: therapeutic targets and strategies. Exp Mol Med.

[54] Bingol B, Sheng M (2011). Deconstruction for reconstruction: the role of proteolysis in neural plasticity and disease. Neuron.

[55] Lago SG, Bahn S (2022). The druggable schizophrenia genome: from repurposing opportunities to unexplored drug targets. NPJ Genom Med.

[56] Schoeler T, Speed D, Porcu E, Pirastu N, Pingault JB, Kutalik Z (2023). Participation bias in the UK Biobank distorts genetic associations and downstream analyses. Nat Hum Behav.

[57] Gkatzionis A, Burgess S (2019). Contextualizing selection bias in Mendelian randomization: how bad is it likely to be?. Int J Epidemiol.

