# Supplementary material for: Circulating Blood-based Proteins in Psychopathology and Cognition: A Mendelian Randomization Study
Source: JAMA Psychiatry. Author manuscript; Available in PMC 2025 Oct 13. (PMC11904806; doi:10.1001/jamapsychiatry.2025.0033)
Supplement: Supplementary material [file EMS207594-supplement-Supplementary_material.docx]

#### **Supplemental Online Content**

Bhattacharyya U, John J, Lam M, et al. Circulating blood-based proteins in psychopathology and cognition: A mendelian randomization study. *JAMA Psychiatry*

##### **eMethods eResults eReferences**

**eAppendix 1.** Data and Code Availability

**eAppendix 2.** eTable References

##### **eFigures**

This supplementary material has been provided by the authors to give readers additional information about their work.

##### **eMethods**

1. **Data Sources and Study Design**

##### **Exposure Data Selection**

In this study, we employed protein quantitative trait loci (pQTL) data from two genome- wide association studies (GWAS) conducted among individuals of European descent to curate genetic instruments for Mendelian randomization analysis.

##### **UK Biobank Pharma Proteomics Project (UKB-PPP)**

This project involves profiling plasma pQTL data for 2,923 proteins derived using the Olink Explore 3072 platform from a cohort of 34,557 UK Biobank participants^1^ of European ancestry. As described in the source publication^1^, pQTL analyses were conducted after controlling for genotyping batch, UKB recruitment centers, UKB array type, UKB-PPP subcohort, and 20 genetic principal components, along with the time between blood sampling and protein measurement.

##### **deCODE Genetics**

In addition, we utilized plasma pQTL data obtained from deCODE Genetics, a study that includes 4,719 proteins measured across 35,559 Icelandic individuals. These proteins were quantified using the SOMAscan version 4 assay^2^. As described in the source publication^2^, pQTL analyses were adjusted for age, sex, and sample age. Mixed models were implemented in BOLT-LMM to control for cryptic relatedness and population stratification, and then LD score regression was utilized in this context to account for inflation in test statistics.

##### Quality Control and Processing of pQTL Dataset

Summary statistics from UKB-PPP and deCODE genetics underwent a QC-filtering process, which involved the exclusion of insertion-deletion (INDELs) variants, variants with a minor allele frequency (MAF) below 0.001, palindromic variants with MAF exceeding 0.42, and, for trans-pQTLs, variants in the extended MHC region (Chr6:25-35Mb). QC-filtered summary statistics were then converted to GWAS VCF files, where the alternative allele denotes the effect allele, using the *gwas2vcf* tool (github.com/MRCIEU/gwas2vcf)^3^. After the QC-filtering step, we selected variants that achieve genome-wide significance (*P* < 5 × 10^−8^) for LD pruning to identify strong, independent genetic instruments. We first separated QC-filtered genome-wide significant variants into cis and trans-pQTLs, where cis-pQTLs are variants within 1 Mb upstream and downstream of the associated protein-coding genes (Ensembl 108 annotations) and QC-filtered genome-wide significant variants outside of the cis region are considered trans- pQTLs. LD pruning was then performed for cis and trans-pQTLs separately to identify independent genetic instruments, using the IEUGWASR tool (Options- Clump_kb=10000, Clump_r2=0.01) with UKB-PPP (N=33,000) as LD reference panel (https://github.com/MRCIEU/ieugwasr).

Our MR instruments included 17,153 cis-pQTLs linked to 2,020 unique proteins assessed within the UKB-PPP cohort (eTable 1a-b) and 23,350 cis-pQTLs related to 1,616 unique proteins measured in the deCODE cohort (eTable 1a-b). Although 818 proteins were assayed on both platforms, yielding a total of 2,818 unique proteins examined in MR using cis- pQTL instruments, we maintained the full denominator of 3,636 analyses for our Bonferroni correction to conservatively account for the fact that we took the lowest available p-value.

Separately, we utilized (as instruments for MR analysis) 31,781 trans-pQTL instruments (excluding the extended MHC region) corresponding to 2,541 unique proteins assessed within the UKB-PPP cohort (eTable 1c-d), as well as 71,747 trans-pQTLs related to 4,175 unique proteins measured in the deCODE cohort (eTable 1c-d). Among these, 1,491 unique proteins

were assayed by both platforms. Despite this overlap, we conservatively controlled for 6,811 tests in our Bonferroni corrections of the trans-pQTL MR results.

##### **Outcome Data**

- - 1. **Psychiatric GWAS Data**

We have obtained the most recent large-scale meta-analyses of GWASs conducted by the Psychiatric Genomics Consortium (PGC) for three complex psychiatric disorders which include schizophrenia (Ncase = 67,323, Ncontrol = 93,456; file name: daner_PGC_SCZ_w3_90_0418b_ukbbdedupe.trios)^4^, bipolar disorder (Ncase = 40,463, Ncontrol = 313,436; file name: daner_bip_pgc3_nm_noukbiobank.gz)^5^, and major depressive disorder (Ncase = 166,773, Ncontrol = 507,679; file name: daner_MDDwoBP_20201001_2015iR15iex_HRC_MDDwoBP_iPSYCH2015i_Wray_FinnGen_ MVPaf_2_HRC_MAF01.gz)^6^ as our outcome data for the MR analysis. The downloaded summary statistics for each disorder included only subjects of European ancestry. They also excluded any subjects drawn from the UK Biobank to avoid any overlap with the cohort that used data from UKB-PPP. Additionally, the SCZ and MDD cohorts did not include subjects that overlapped with the deCODE pQTL cohort. However, the GWAS of bipolar disorder contained samples from deCODE (Ncase = 1972, Ncontrol = 192,602); summary statistics excluding these individuals were not available, and therefore we performed statistical tests to determine if substantial overlap was present (see Section 4 below). As described in the original publications, all psychiatric GWAS were performed using principal components analysis to control for effects of population stratification.

##### **Cognitive Task Performance (CTP) GWAS Data**

Separately, for Cognitive Task Performance (CTP), we have recomputed GWAS for the largest available sample size of European ancestry subjects, excluding individuals in the UKB-

PPP cohort. (It should be noted that this GWAS also contains no individuals from deCODE.) Specifically, we first accessed the genotypic data for “fluid intelligence” (Field ID: 20016) from the UK Biobank (UKB) cohort for all individuals of European ancestry, excluding those assayed on the proteomic platform of UKB-PPP (remaining N=141,123). Our analysis comprised the following key steps: initial sample and genotype quality control, excluding variants with genotyping call rates below 90%, those in Hardy-Weinberg disequilibrium (PHWE < 1 x 10^−15^), and variants with a minor allele frequency <1%. We then performed a GWAS of the “fluid intelligence” phenotype using the REGENIE pipeline (version 3.2.4)^7^, with covariates including sex, mean age, age^2^, sex by age interaction, sex by age^2^ interaction, and the top 20 principal components.

Subsequently, we meta-analyzed these results with GWAS summary statistics from the cognitive GWAS of Savage et al. (2018)^8^, excluding all subjects from the UK Biobank (remaining N=74,210; file name: Cognition_Meta_GWAS_without_UKBPP1.tbl). GWAS meta-analysis was performed using METAL software^9^. The resulting GWAS summary statistics (N = 215,333) were employed as outcome data for the CTP trait in our MR analysis.

##### Outcome Data for Potential Confounding Phenotypes

We obtained the most recent large-scale meta-analyses of GWASs focused on smoking and alcohol usage phenotypes, specifically representing the amount smoked and alcohol consumption. For alcohol use, we selected a GWAS measured as drinks per week (DrnkWk; n = 2,965,643)^10^. Additionally, we used a GWAS on smoking quantity among current and former regular smokers, measured as cigarettes smoked per day (CigDay; n = 784,353)^10^. Both the GWAS were without any UKBB samples. To control for potential pleiotropic confounds with these phenotypes, we removed any of the significant instrumental variables in either of these GWASs and performed our primary MR analyses again. To further test for potential pleiotropic

effects of non-psychiatric diseases, we performed a phenomewide association study on all nominally significant (P<0.05) SNP instruments within all Bonferroni-significant genes emerging from our MR analyses, using traits drawn from the IEU OpenGWAS database, filtered using the keywords “cancer” and “disease.”

##### **Processing the GWAS Summary Statistics**

Summary statistics data of the three psychiatric GWASs and CTP meta-GWAS were converted to a GWAS VCF file using the gwas2vcf tool. To ensure consistency of genomic coordinates all the psychiatric GWAS summary statistics were converted into the GRCh38 build to match that of the pQTL data.

##### **Informed Consent**

Study participants for all GWAS utilized in the present study, including both exposure datasets (deCODE and UK Biobank) and all outcome datasets, provided written informed consent to protocols approved by local institutional review boards, as detailed in the original GWAS publications.

##### Mendelian Randomization (MR) Analysis

We performed MR analyses using the ‘MendelianRandomization’ package version 0.8.0^11,12^ within the R environment. SNPs significantly associated with each protein (i.e., pQTLs) were used as instruments. The package includes approaches that compute Wald’s ratio for each SNP instrument’s effect on the outcome phenotype; when multiple SNP instruments are available for a given protein, these effects are combined using the delta-weighted inverse- variance method (IVW-Delta). We applied default parameters for ‘MendelianRandomization’, where the function that estimates fixed-effects IVW-Delta was carried out only when fewer than four SNP instruments were available; otherwise, multiplicative random-effects IVW-Delta was

employed. Random-effects meta-analysis is more conservative than fixed-effects meta-analysis in the presence of heterogeneity across instruments without sacrificing power in the absence of heterogeneity^13^. Moreover, multiplicative random-effect meta-analysis is more conservative in the presence of pleiotropy^14^ than an additive random-effects approach. (For comparison purposes and as a technical replication, we also performed MR analyses with standard fixed- effects IVW, using the commonly utilized TwoSampleMR package^15^; results of this comparison are available in eTable 2.)

MR analyses were conducted separately for each pQTL dataset (UKB-PPP and deCODE). For the proteins that are present in both UKB-PPP and deCODE data, we conducted a p-value-based meta-analysis using Fisher’s method implemented in the metap package (https://github.com/cran/metap/blob/master/R/sumlog.R; sumlog function). Analyses were also conducted separately for cis-pQTLs and trans-pQTLs excluding the extended MHC region (which will be referred to simply as trans-pQTLs in subsequent text).

##### **Horizontal Pleiotropy and Heterogeneity Tests**

To test robustness of our results and sensitivity to potential confounds, we have used the MR-Egger intercept test^16^ from the TwoSampleMR package^15^ to estimate horizontal pleiotropy, and Cochran’s *Q* statistic derived from the IVW analysis to estimate heterogeneity^17^. Importantly, while there is no universally accepted gold standard amongst MR approaches, we selected the more conservative options available throughout this work. Therefore, our primary method of determining significance for MR results utilized IVW-Delta, multiplicative random effects in the ‘MendelianRandomization” package^11,12^, which reduces impact of outlier values in the face of instrument heterogeneity. By contrast, many proteins demonstrated inflated significance levels in our complementary/sensitivity analyses using fixed-effects IVW implemented in the TwoSampleMR software (see eTable 2). Moreover, multiple proteins that

would have been deemed statistically significant by fixed-effects IVW in TwoSampleMR did not attain significance in our analysis, and are therefore not included in our reported results.

##### **Reverse Causal Directionality Test**

To confirm the “correct” directionality of effects (i.e., that the exposure causes the outcome rather than the reverse), we employed the MR Steiger test, as implemented in the TwoSampleMR package (although it is acknowledged that the MR Steiger test can only detect strong violations of the instrumental variable assumptions). Any FDR significant proteins (FDR<0.05), both from cis and trans findings, that were suggested by the MR Steiger test to potentially have the incorrect causal direction, were then tested for reverse causal directionality in a second MR analysis using SCZ, BIP, MDD, or CTP as the exposure phenotype and UKBB- pQTL or deCODE-pQTL as the outcome phenotype.

##### **Sensitivity Analysis using Robust MR Methods**

We employed several Mendelian Randomization (MR) methods^12^ to test the robustness of our findings, focusing on those sensitive to weak instrument bias and pleiotropic outliers.

Here, "pleiotropy" is used in a statistical genetics sense, not a biological one. Our primary sensitivity analyses were the commonly used MR-PRESSO as well as median- and mode- based methods, although we also employed less commonly used approaches such as the contamination mixture method and constrained maximum likelihood (MR-cML)^12^. Each method operates under different assumptions to provide causal estimates, allowing us to evaluate consistency of results across varying potential confounds. MR-PRESSO refines the IVW method by removing variants with significantly different causal estimates before performing the IVW analysis on the remaining variants. MR-cML is particularly robust, allowing for violations of any of the three instrumental variable assumptions and offering better inference through data perturbation. The contamination mixture method also has a plurality-valid assumption and is

designed to handle situations with pleiotropic variants. MR-Egger-Robust, on the other hand, assumes that pleiotropic effects are independent of variant-exposure associations, known as the Instrument Strength Independent of Direct Effect (InSIDE) assumption^12^. It is particularly sensitive to outliers and can produce imprecise estimates, especially when the variant-exposure associations are similar in magnitude. The median-based method assumes that less than half of the genetic variants are invalid instruments (majority valid assumption). It is robust to outliers and provides consistent estimates even when some instruments are invalid. Mode-based methods operate assuming that the largest group of variants estimates the true causal effect (plurality valid assumption)^12^.

##### **Multiple Correction**

We applied Bonferroni correction separately for cis-pQTL and trans-pQTL analyses within each phenotype. We further performed Benjamini-Hochberg False Discovery Rate (FDR) correction on the lowest p-values obtained from the UKB-PPP, deCODE, or metap for cis- and trans-pQTL instruments, separately for all four phenotypes. Specifically, a total of 3636 proteins had cis-pQTL instruments, and 6716 proteins had trans-pQTL instruments available, resulting in a Bonferroni-corrected p-value (*P*BONF) of 1.3x10^-5^ (0.05/3,636) for Mendelian Randomization analysis of proteins using cis-pQTLs, and 7.4x10^-6^ (0.05/6,710) for MR analysis of proteins with trans-pQTLs. Please note that the results from the sensitivity tests for each protein in every analysis have been reported based on the dataset (either UKBB or deCODE) that yielded the lower P value.

##### **Sample Overlap Between Cohort Used in deCODE pQTL Study and BIP**

To check if control populations utilized in the BIP GWAS cohort overlap with the cohort used by deCODE to generate pQTL data, we performed LD Score regression intercept analysis for the top 20 proteins from our significant findings. The LD Score regression covariate intercept

between BIP and deCODE pQTL was no higher than 0.016 in all cases. Thus, we can assume that the sample overlap between BIP and deCODE pQTL cohorts was negligible. Furthermore, the majority of our significant findings for BIP were observed in the UKB-PPP protein dataset, which does not share any overlap with the bipolar disorder GWAS cohort.

##### **Systematic Literature Review**

To determine the novelty of our findings, we conducted a comprehensive and systematic literature review using the "advanced search" feature on PubMed, encompassing all outcome phenotypes considered in our study. Studies examining molecular traits in relation to psychiatric GWAS have primarily focused on gene expression quantitative trait loci (eQTLs), so these were included in our search along with studies of pQTLs. (It should be noted that transcriptomic and proteomic data can often differ^18^.) Moreover, such studies have frequently examined brain- based molecular traits, which have the advantage of direct assessment of neural tissue.

However, brain-based studies have the disadvantage of relatively small sample sizes compared to our pQTL reference panels of ~35,000 individuals each. Thus, our search strategy on PubMed was as follows:

Include the following terms in their title or abstracts: ("pqtl" or "pqtls" or "expression quantitative trait loci" or "protein quantitative trait loci" or "eqtl" or "eqtls" or "proteome" or "proteomics" or "proteomic" or "transcriptome" or "transcriptomic" or "transcriptomics" or "qtl" or "proteomes" or “transcriptomes" or "proteogenomic" or "expression") AND ("TWAS" or "transcriptome-wide association study" or "transcriptome wide association study" or "SMR" or "Summary data based mendelian randomization" or "mendelian randomization" or "MR" or "Mendelian randomisation" or "cis").

The initial search resulted in 43,226 publications found. We then refined our search within the 43,226 publications that include any one or more following terms in their title:

"schizophrenia" or "bipolar” or "depression" or "depressive" or "cognition" or "cognitive" or "intelligence" or "education" or "psychiatric" or "neurological" or "common disorders" or "common diseases" or "common trait" or "complex phenotypes" or "complex traits" or "complex trait" or "complex disorders" or "complex diseases" or "brain" or "neuropsychiatric".

This refinement resulted in 998 publications. We automatically (within PubMed) removed any reviews and papers without human data and manually removed papers with data from induced pluripotent stem cells or organoids or tissues other than brain or blood, papers without available data, and preprints. A total of 50 relevant publications remained; these selected articles were thoroughly reviewed to evaluate the novelty of our work. The novelty of our findings was evaluated using two distinct thresholds. Proteins deemed significant at the stringent Bonferroni level (*P*BONF < 0.05) in our study were classified as novel if they lacked prior investigation or exhibited a P-value > 0.000015 or a PP4 interaction score less than 0.7 in the included literature. Similarly, proteins reaching significance at the less stringent 5% false discovery rate threshold (FDR < 0.05) in our study were categorized as novel if there was no prior investigation on them or if they displayed a P-value > 0.001 or a PP4<0.7 within the included studies.

##### **Pathway Enrichment**

We performed gene-set enrichment analysis, examining only those proteins identified with the highest confidence level: specifically, proteins that were strictly significant (*P*BONF < 0.05) in the cis-pQTL analyses for each phenotype. We used a web-based tool, EnrichR^19^, specifically using its integrated gene-set libraries from KEGG, Reactome, MSigDB, and Gene Ontology (Cellular component, Molecular Function, and Biological Process).

##### Protein-protein Interaction

For these same Bonferroni-significant proteins emerging from the cis-pQTL analyses, a protein-protein interaction network for each outcome phenotype was constructed using the STRING database (https://string-db.org/)^20^. This online tool incorporates a broad range of data sources to derive both known and predicted protein-protein interactions from both direct (physical) and indirect (functional) associations. Network images are derived using a spring model, with nodes modeled as masses and edges as springs; the final position of the nodes in the image is computed by minimizing the 'energy' of the system.

##### **Drug Target Enrichment**

Further, for these same Bonferroni-significant proteins emerging from the cis-pQTL analyses, we performed a Drug Target enrichment analysis in EnrichR^19^, specifically using its integrated libraries from DSigDB^21^, DrugMatriX, and CMAP.

To further examine the druggability of these significant proteins emerging from Mendelian Randomization, we utilized DGIdb5.0^22^ (www.dgidb.org) to identify approved compounds, immunotherapies, and known chemical compounds that could interact with these proteins. Additionally, we used DGIdb5.0 to identify potential druggable genes that are known or predicted to interact with drugs. DGIdb is an online tool that uses a combination of expert curation and text-mining to extract drug-gene interactions from resources such as DrugBank, PharmGKB, ChEMBL, Drug Target Commons. Furthermore, DGIdb can categorize genes as potentially druggable based on their presence in selected pathways, molecular functions, and gene families from the Gene Ontology, the Human Protein Atlas, IDG, "druggable genome" lists from Hopkins and Groom^23^and Russ and Lampel^24^, among others.

##### **eResults**

*Results using the FDR threshold.*

We employed a less strict, 5% false discovery rate (FDR<0.05) correction on the lowest p-values obtained from the UKB-PPP, deCODE, or metap for cis- and trans-pQTL instruments, separately for all four phenotypes. A total of 213 proteins were predicted to have a causal association with susceptibility to psychiatric disorders and/or cognitive performance in MR analysis using cis-pQTLs as the MR instruments. Specifically, 129 proteins were genetically predicted to have causal associations with SCZ, 55 with BIP, 15 with MDD, and 74 with CTP (Table 1, eTable 2). Our literature search revealed that a large proportion of these associations were not previously reported as significant (at FDR<0.05); specifically, 52 of these proteins were novel for SCZ, 18 for BIP, 13 for MDD, and 53 were novel for CTP (Table1, eTable 3a-c).

Except for 8 proteins (SCZ:7; CTP:1), all FDR-significant proteins were significant for “true” directionality. In the reverse MR analyses for these proteins, MR-Steiger test indicated that the reverse direction was not “true” for any of these. Additionally, only one associated protein was found to have nominally significant (*P* < 0.05) horizontal pleiotropy. Using Cochran’s *Q* statistic derived from the IVW meta-analysis, 8 proteins in SCZ, 3 proteins in BIP, and 5 proteins in CTP were found to have nominally significant (*P* < 0.05) heterogeneity (for forest plots, see eFigures 3A-3BK). However, as noted previously, our use of multiplicative random- effects IVW provides conservative p-values in the presence of such heterogeneity. Among the 213 proteins that were found to be associated at the looser threshold (FDR < 0.05), 79 proteins were assayed in both cohorts, and all associations were replicated in both cohorts (44 for SCZ, 24 for BIP, 7 for MDD, and 29 for CTP). By contrast, 90 (55 for SCZ, 20 for BIP, 6 for MDD, and 30 for CTP) and 44 (30 for SCZ, 11 for BIP, 2 for MDD, and 15 for CTP) unique proteins were specifically assayed only in UKB-PPP and deCODE cohort respectively (eTable 2).

In our additional sensitivity analysis utilizing MR-PRESSO, all findings remained significant. In MR-IVW Robust, except for 6 proteins (SCZ:1, BIP:2, CTP:3), all remained significant (eTable 2a). In each of the remaining sensitivity analyses, the large majority of proteins retained significance, except: 5 (BIP:3, CTP:2) proteins in MRConMix, 5 (SCZ:2, BIP:3, CTP:2) proteins in MRcML, 17 (SCZ:2, BIP:3, CTP:12) in the weighted median test, 38 (SCZ:20, BIP: 7, MDD: 2, CTP:9) in the weighted mode test, and 17 (SCZ:2, BIP:3, CTP:12) in MR Egger Robust (eTable 2a).

In trans-pQTL MR analyses, associations with a total of 51 proteins were identified for the outcome phenotypes (Table1, eTable 2) at the less stringent threshold (FDR < 0.05).

Specifically, 27 proteins were associated with SCZ, 7 with BIP, 3 with MDD, and 31 with CTP. Our review of the literature on prior blood- or brain-based TWAS, SMR, and MR analysis revealed that 21 findings for SCZ, 6 for BIP, 1 for MDD, and 28 for CTP were novel. In Steiger’s directionality test, 6 proteins for CTP, 10 for SCZ, 2 for MDD, and 1 for BIP were not statistically significant (although indicated as “true” for correct directionality). However, when we performed reverse causality for these proteins, all except for one (RYR1) were identified as “false” in directionality, with a significant P value in Steiger’s directionality test.

Amongst trans-pQTL proteins identified using the FDR < 0.05 threshold, 5 proteins that were associated with CTP, 8 proteins associated with SCZ, and 3 proteins associated with BIP were found to have at least nominally significant (*P* < 0.05) heterogeneity (eTable 2). Among the trans-pQTL proteins (FDR < 0.05), only 21 proteins (10 for SCZ, 4 for BIP, and 8 for CTP) were assayed in both platforms (eTable 2) and their association were replicated in both cohorts. It is important to note, however, that the overall set of MR results derived from trans-pQTL instruments were only weakly correlated across platforms, suggesting that caution should be applied in interpreting these results.

In our additional sensitivity analysis utilizing MR-PRESSO, all findings remained significant except for HBEGF (CTP). All the findings remained significant in MR-IVW Robust, Weighted median and MRcML. However, 4 proteins (SCZ:3, CTP:1) did not remain significant in Robust MR-Egger and 1 protein was not significant in MRConMix. In weighted mode analysis, 6 proteins and 8 proteins did not retain significance for CTP and SCZ respectively. However, only one protein (HBEGF) did not retain significance for CTP in more than one sensitivity analyses.

*Enrichment analyses for genes significant for multiple outcomes.*

In the pathway enrichment analysis with genes that were strictly significant (PBONF < 0.05) in more than one disorder, neurodevelopment-related pathways such as neuron projection development and regulation of cellular growth were found to be significant. For genes that were FDR-significant in more than one disorder, enrichment in immune related pathways such as

Toll-like receptor signaling pathway, MHC Class II Antigen Presentation in addition to neurodevelopmental pathways were observed. In the pathway enrichment analysis with disease-specific genes, we observed enrichment of pathways similar to those observed with all the significant genes from each phenotype.

The trans-pQTL analysis did not yield a sufficient number of genes common across multiple disorders to perform pathway enrichment analysis. The number of significant genes specific to other phenotypes was not sufficient to perform enrichment analyses, with the exception of CTP; pathway enrichment analyses with phenotype-specific genes showed enrichment in regulation of cell population related pathways.

##### **eReferences**

1. Sun, B. B. *et al.* Plasma proteomic associations with genetics and health in the UK Biobank. *Nature* **622**, 329–338 (2023).
2. Ferkingstad, E. *et al.* Large-scale integration of the plasma proteome with genetics and disease. *Nat. Genet.* **53**, 1712–1721 (2021).
3. Lyon, M. S. *et al.* The variant call format provides efficient and robust storage of GWAS summary statistics. *Genome Biol.* **22**, 32 (2021).
4. Trubetskoy, V. *et al.* Mapping genomic loci implicates genes and synaptic biology in schizophrenia. *Nature* **604**, 502–508 (2022).
5. Mullins, N. *et al.* Genome-wide association study of more than 40,000 bipolar disorder cases provides new insights into the underlying biology. *Nat. Genet.* **53**, 817–829 (2021).
6. Als, T. D. *et al.* Depression pathophysiology, risk prediction of recurrence and comorbid psychiatric disorders using genome-wide analyses. *Nat. Med.* **29**, 1832–1844 (2023).
7. Mbatchou, J. *et al.* Computationally efficient whole-genome regression for quantitative and binary traits. *Nat. Genet.* **53**, 1097–1103 (2021).
8. Savage, J. E. *et al.* Genome-wide association meta-analysis in 269,867 individuals identifies new genetic and functional links to intelligence. *Nat. Genet.* **50**, 912–919 (2018).
9. Willer, C. J., Li, Y. & Abecasis, G. R. METAL: fast and efficient meta-analysis of genomewide association scans. *Bioinformatics* **26**, 2190–2191 (2010).
10. Saunders, G. R. B. *et al.* Genetic diversity fuels gene discovery for tobacco and alcohol use. *Nature* **612**, 720–724 (2022).
11. Yavorska, O. O. & Burgess, S. MendelianRandomization: an R package for performing Mendelian randomization analyses using summarized data. *Int. J. Epidemiol.* **46**, 1734– 1739 (2017).
12. Burgess, S. *et al.* Guidelines for performing Mendelian randomization investigations: update for summer 2023. *Wellcome Open Res* **4**, 186 (2019).
13. Li, X., Jiang, L., Xue, C., Li, M. J. & Li, M. A conditional gene-based association framework integrating isoform-level eQTL data reveals new susceptibility genes for schizophrenia. *Elife* **11**, (2022).
14. Bowden, J. *et al.* A framework for the investigation of pleiotropy in two-sample summary data Mendelian randomization. *Stat. Med.* **36**, 1783–1802 (2017).
15. Hemani, G. *et al.* The MR-Base platform supports systematic causal inference across the human phenome. *Elife* **7**, (2018).
16. Burgess, S. & Thompson, S. G. Interpreting findings from Mendelian randomization using the MR-Egger method. *Eur. J. Epidemiol.* **32**, 377–389 (2017).
17. Burgess, S., Bowden, J., Fall, T., Ingelsson, E. & Thompson, S. G. Sensitivity Analyses for Robust Causal Inference from Mendelian Randomization Analyses with Multiple Genetic Variants. *Epidemiology* **28**, 30–42 (2017).
18. Buccitelli, C. & Selbach, M. mRNAs, proteins and the emerging principles of gene expression control. *Nat. Rev. Genet.* **21**, 630–644 (2020).
19. Kuleshov, M. V. *et al.* Enrichr: a comprehensive gene set enrichment analysis web server 2016 update. *Nucleic Acids Res.* **44**, W90-7 (2016).
20. Szklarczyk, D. *et al.* The STRING database in 2023: protein-protein association networks and functional enrichment analyses for any sequenced genome of interest. *Nucleic Acids Res.* **51**, D638–D646 (2023).
21. Yoo, M. *et al.* DSigDB: drug signatures database for gene set analysis. *Bioinformatics* **31**, 3069–3071 (2015).
22. Cannon, M. *et al.* DGIdb 5.0: rebuilding the drug-gene interaction database for precision medicine and drug discovery platforms. *Nucleic Acids Res.* **52**, D1227–D1235 (2024).
23. Hopkins, A. L. & Groom, C. R. The druggable genome. *Nat. Rev. Drug Discov.* **1**, 727–730 (2002).
24. Russ, A. P. & Lampel, S. The druggable genome: an update. *Drug Discov. Today* **10**, 1607–1610 (2005)

**eAppendix 1. Data and Code Availability**

Outcome GWAS summary statistics can be downloaded from:

SCZ: received from PGC consortium - will be made available upon publication BIP: https://Figureshare.com/ndownloader/files/40036705

MDD:https://ipsych.dk/fileadmin/ipsych.dk/Downloads/daner_MDDwoBP_20201001_2015iR1 5iex_HRC_MDDwoBP_iPSYCH2015i_Wray_FinnGen_MVPaf_2_HRC_MAF01.gz

CTP: Received from UKBB - will be made available upon publication

CigDay without UKBB: https://conservancy.umn.edu/bitstreams/d50f9dc8-df08-48a3-9586- f5627281ab2d/download

DrnkWk without UKBB: https://conservancy.umn.edu/bitstreams/b9a3372f-3ab6-4b09-a63b- 13a6a8376a9b/download

Analytic software packages utilized:

- 1. Mendelian Randomization (MR) package

(GitHub link: https://github.com/cran/MendelianRandomization )

- 1. TwoSampleMR package

(GitHub link: https://mrcieu.github.io/TwoSampleMR/index.html )

- 1. Meta-analysis package

(Github link: https://github.com/cran/metap/blob/master/R/sumlog.R)

- 1. EnrichR

(Webportal link: https://maayanlab.cloud/Enrichr/)

- 1. StringDB - Protein-Protein Interaction (Webportal link: https://string-db.org/ )
  2. DGIdb - Protein-Protein Interaction (Webportal link: https://dgidb.org/ )
  3. IEU OpenGWAS API

(Github link: https://github.com/MRCIEU/ieugwasr)

**eAppendix 2. eTable References**

1. Dang, X., Song, M., Lv, L., Yang, Y. & Luo, X.-J. Proteome-wide Mendelian randomization reveals the causal effects of immune-related plasma proteins on psychiatric disorders. *Hum. Genet.* **142**, 809–818 (2023).
2. Lu, T., Forgetta, V., Greenwood, C. M. T., Zhou, S. & Richards, J. B. Circulating Proteins Influencing Psychiatric Disease: A Mendelian Randomization Study. *Biol. Psychiatry* **93**, 82–91 (2023).
3. Deng, Y.-T. *et al.* Identifying causal genes for depression via integration of the proteome and transcriptome from brain and blood. *Mol. Psychiatry* **27**, 2849–2857 (2022).
4. Gandal, M. J. *et al.* Transcriptome-wide isoform-level dysregulation in ASD, schizophrenia, and bipolar disorder. *Science* **362**, (2018).
5. Gedik, H. *et al.* Identifying potential risk genes and pathways for neuropsychiatric and substance use disorders using intermediate molecular mediator information. *Front. Genet.* **14**, 1191264 (2023).
6. Hall, L. S. *et al.* A transcriptome-wide association study implicates specific pre- and post- synaptic abnormalities in schizophrenia. *Hum. Mol. Genet.* **29**, 159–167 (2020).
7. Jiang, L. *et al.* Powerful and robust inference of complex phenotypes’ causal genes with dependent expression quantitative loci by a median-based Mendelian randomization. *Am. J. Hum. Genet.* **109**, 838–856 (2022).
8. Wingo, T. S. *et al.* Brain proteome-wide association study implicates novel proteins in depression pathogenesis. *Nat. Neurosci.* **24**, 810–817 (2021).
9. Huang, Y. *et al.* Patterns of Convergence and Divergence Between Bipolar Disorder Type I and Type II: Evidence From Integrative Genomic Analyses. *Front Cell Dev Biol* **10**, 956265 (2022).
10. Benjamin, K. J. M. *et al.* Analysis of the caudate nucleus transcriptome in individuals with schizophrenia highlights effects of antipsychotics and new risk genes. *Nat. Neurosci.* **25**, 1559– 1568 (2022).
11. Aygün, N. *et al.* Brain-trait-associated variants impact cell-type-specific gene regulation during neurogenesis. *Am. J. Hum. Genet.* **108**, 1647–1668 (2021).
12. Collado-Torres, L. *et al.* Regional Heterogeneity in Gene Expression, Regulation, and Coherence in the Frontal Cortex and Hippocampus across Development and Schizophrenia. *Neuron* **103**, 203–216.e8 (2019).
13. Walker, R. L. *et al.* Genetic Control of Expression and Splicing in Developing Human Brain Informs Disease Mechanisms. *Cell* **179**, 750–771.e22 (2019).
14. Wingo, T. S. *et al.* Shared mechanisms across the major psychiatric and neurodegenerative diseases. *Nat. Commun.* **13**, 4314 (2022).
15. Hauberg, M. E. *et al.* Large-Scale Identification of Common Trait and Disease Variants Affecting Gene Expression. *Am. J. Hum. Genet.* **100**, 885–894 (2017).
16. He, Q. *et al.* Non-steroidal anti-inflammatory drug target gene associations with major depressive disorders: a Mendelian randomisation study integrating GWAS, eQTL and mQTL Data. *Pharmacogenomics J.* **23**, 95–104 (2023).
17. Wu, Y., Yu, X.-L., Xiao, X., Li, M. & Li, Y. Joint-Tissue Integrative Analysis Identified Hundreds of Schizophrenia Risk Genes. *Mol. Neurobiol.* **59**, 107–116 (2022).
18. Chauquet, S. *et al.* Association of Antihypertensive Drug Target Genes With Psychiatric Disorders: A Mendelian Randomization Study. *JAMA Psychiatry* **78**, 623–631 (2021).
19. Bryois, J. *et al.* Cell-type-specific cis-eQTLs in eight human brain cell types identify novel risk genes for psychiatric and neurological disorders. *Nat. Neurosci.* **25**, 1104–1112 (2022).
20. Li, X., Shen, A., Zhao, Y. & Xia, J. Mendelian Randomization Using the Druggable Genome Reveals Genetically Supported Drug Targets for Psychiatric Disorders. *Schizophr. Bull.* **49**, 1305–1315 (2023).
21. Dang, X., Liu, J., Zhang, Z. & Luo, X.-J. Mendelian Randomization Study Using Dopaminergic Neuron-Specific eQTL Identifies Novel Risk Genes for Schizophrenia. *Mol. Neurobiol.* **60**, 1537–1546 (2023).
22. Liu, J., Li, X. & Luo, X.-J. Proteome-wide Association Study Provides Insights Into the Genetic Component of Protein Abundance in Psychiatric Disorders. *Biol. Psychiatry* **90**, 781– 789 (2021).
23. Gu, X. *et al.* Identifying novel proteins underlying schizophrenia via integrating pQTLs of the plasma, CSF, and brain with GWAS summary data. *BMC Med.* **20**, 1–10 (2022).
24. Liu, H. *et al.* Integrated Analysis of Summary Statistics to Identify Pleiotropic Genes and Pathways for the Comorbidity of Schizophrenia and Cardiometabolic Disease. *Front. Psychiatry* **11**, 256 (2020).
25. Korologou-Linden, R., Leyden, G. M., Relton, C. L., Richmond, R. C. & Richardson, T. G. Multi-omics analyses of cognitive traits and psychiatric disorders highlights brain-dependent mechanisms. *Hum. Mol. Genet.* **32**, 885–896 (2021).
26. Gusev, A. *et al.* Transcriptome-wide association study of schizophrenia and chromatin activity yields mechanistic disease insights. *Nat. Genet.* **50**, 538–548 (2018).
27. Zhang, C. *et al.* Brain transcriptome-wide association study implicates novel risk genes underlying schizophrenia risk. *Psychol. Med.* 1–11 (2023).
28. Wu, Y., Zhang, C.-Y., Wang, L., Li, Y. & Xiao, X. Genetic Insights of Schizophrenia via Single Cell RNA-Sequencing Analyses. *Schizophr. Bull.* **49**, 914–922 (2023).
29. Huang, J. *et al.* Circulatory proteins relate cardiovascular disease to cognitive performance: A mendelian randomisation study. *Front. Genet.* **14**, 1124431 (2023).
30. de Klein, N. *et al.* Brain expression quantitative trait locus and network analyses reveal downstream effects and putative drivers for brain-related diseases. *Nat. Genet.* **55**, 377–388 (2023).
31. Wu, C. & Pan, W. Integrating eQTL data with GWAS summary statistics in pathway-based analysis with application to schizophrenia. *Genet. Epidemiol.* **42**, 303–316 (2018).
32. Gamazon, E. R., Zwinderman, A. H., Cox, N. J., Denys, D. & Derks, E. M. Multi-tissue transcriptome analyses identify genetic mechanisms underlying neuropsychiatric traits. *Nat. Genet.* **51**, 933–940 (2019).
33. Yang, C.-P. *et al.* Comprehensive integrative analyses identify GLT8D1 and CSNK2B as schizophrenia risk genes. *Nat. Commun.* **9**, 838 (2018).
34. Liu, J. *et al.* Genome-wide Mendelian randomization identifies actionable novel drug targets for psychiatric disorders. *Neuropsychopharmacology* **48**, 270–280 (2023).
35. Mancuso, N. *et al.* Integrating Gene Expression with Summary Association Statistics to Identify Genes Associated with 30 Complex Traits. *Am. J. Hum. Genet.* **100**, 473–487 (2017).
36. Liu, J. *et al.* Genome-wide association study followed by trans-ancestry meta-analysis identify 17 new risk loci for schizophrenia. *BMC Med.* **19**, 177 (2021).
37. Kibinge, N. K., Relton, C. L., Gaunt, T. R. & Richardson, T. G. Characterizing the Causal Pathway for Genetic Variants Associated with Neurological Phenotypes Using Human Brain- Derived Proteome Data. *Am. J. Hum. Genet.* **106**, 885–892 (2020).
38. Gudjonsson, A. *et al.* A genome-wide association study of serum proteins reveals shared loci with common diseases. *Nat. Commun.* **13**, 480 (2022).
39. Li, X. *et al.* Transcriptome-wide association study identifies new susceptibility genes and pathways for depression. *Transl. Psychiatry* **11**, 306 (2021).
40. Zhu, Z. *et al.* Integration of summary data from GWAS and eQTL studies predicts complex trait gene targets. *Nat. Genet.* **48**, 481–487 (2016).
41. Yang, H. *et al.* Mendelian randomization integrating GWAS and eQTL data revealed genes pleiotropically associated with major depressive disorder. *Transl. Psychiatry* **11**, 225 (2021).
42. Dall’Aglio, L., Lewis, C. M. & Pain, O. Delineating the Genetic Component of Gene Expression in Major Depression. *Biol. Psychiatry* **89**, 627–636 (2021).
43. Wang, X. *et al.* Integrating genome-wide association study and expression quantitative trait loci data identifies NEGR1 as a causal risk gene of major depression disorder. *J. Affect. Disord.* **265**, 679–686 (2020).
44. Zhao, B. *et al.* Transcriptome-wide association analysis of brain structures yields insights into pleiotropy with complex neuropsychiatric traits. *Nat. Commun.* **12**, 2878 (2021).
45. Yao, S. *et al.* Epigenetic Element-Based Transcriptome-Wide Association Study Identifies Novel Genes for Bipolar Disorder. *Schizophr. Bull.* **47**, 1642–1652 (2021).
46. Baird, D. A. *et al.* Identifying drug targets for neurological and psychiatric disease via genetics and the brain transcriptome. *PLoS Genet.* **17**, e1009224 (2021).
47. Zhao, Y. *et al.* Integrating genome-wide association study and expression quantitative trait locus study identifies multiple genes and gene sets associated with schizophrenia. *Prog.*

*Neuropsychopharmacol. Biol. Psychiatry* **81**, 50–54 (2018).

1. Png, G. *et al.* Mapping the serum proteome to neurological diseases using whole genome sequencing. *Nat. Commun.* **12**, 7042 (2021).
2. Mei, J. *et al.* The needle in the haystack: Identifying and validating common genes of depression, insomnia, and inflammation. *J. Affect. Disord.* **342**, 45–53 (2023).
3. Wang, J.-Y. *et al.* Integrative Analyses Followed by Functional Characterization Reveal TMEM180 as a Schizophrenia Risk Gene. *Schizophr. Bull.* **47**, 1364–1374 (2021).

**eFigure 1: Detailed MR analysis workflow**

**1A.**

**1B.**

1C.

**eFigure 2.** Manhattan plot showing findings from MR analysis for the outcome phenotypes A. schizophrenia, B. bipolar disorder, C. major depressive disorder, and D. cognitive task performance, employing Cis and Trans-pQTLs (without extended MHC) from UKB-PPP and deCODE dataset as instrumental variables. Please note gene names in red font are novel findings.

**2A. Schizophrenia**


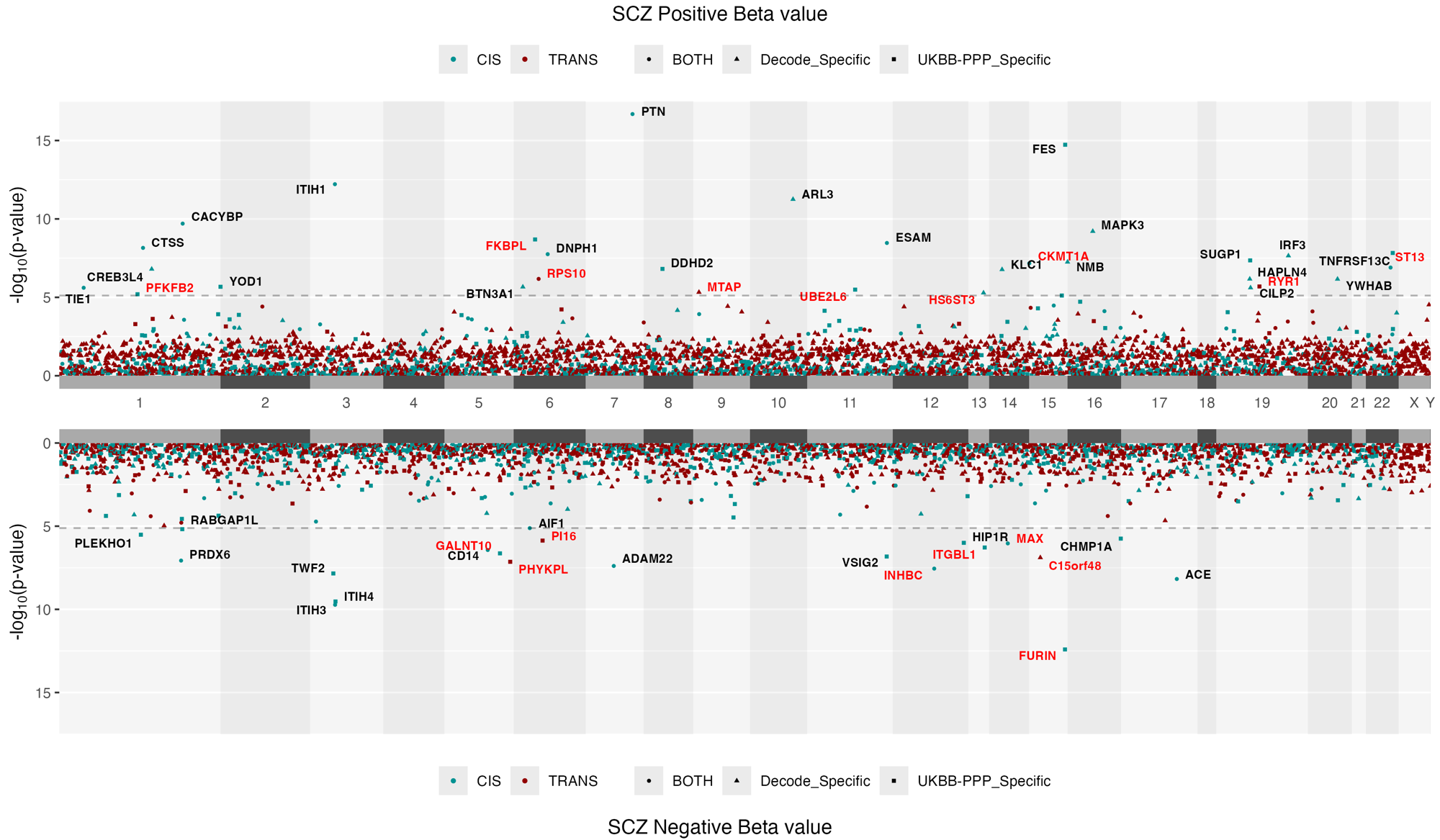


**2B. Bipolar Disorder**

**
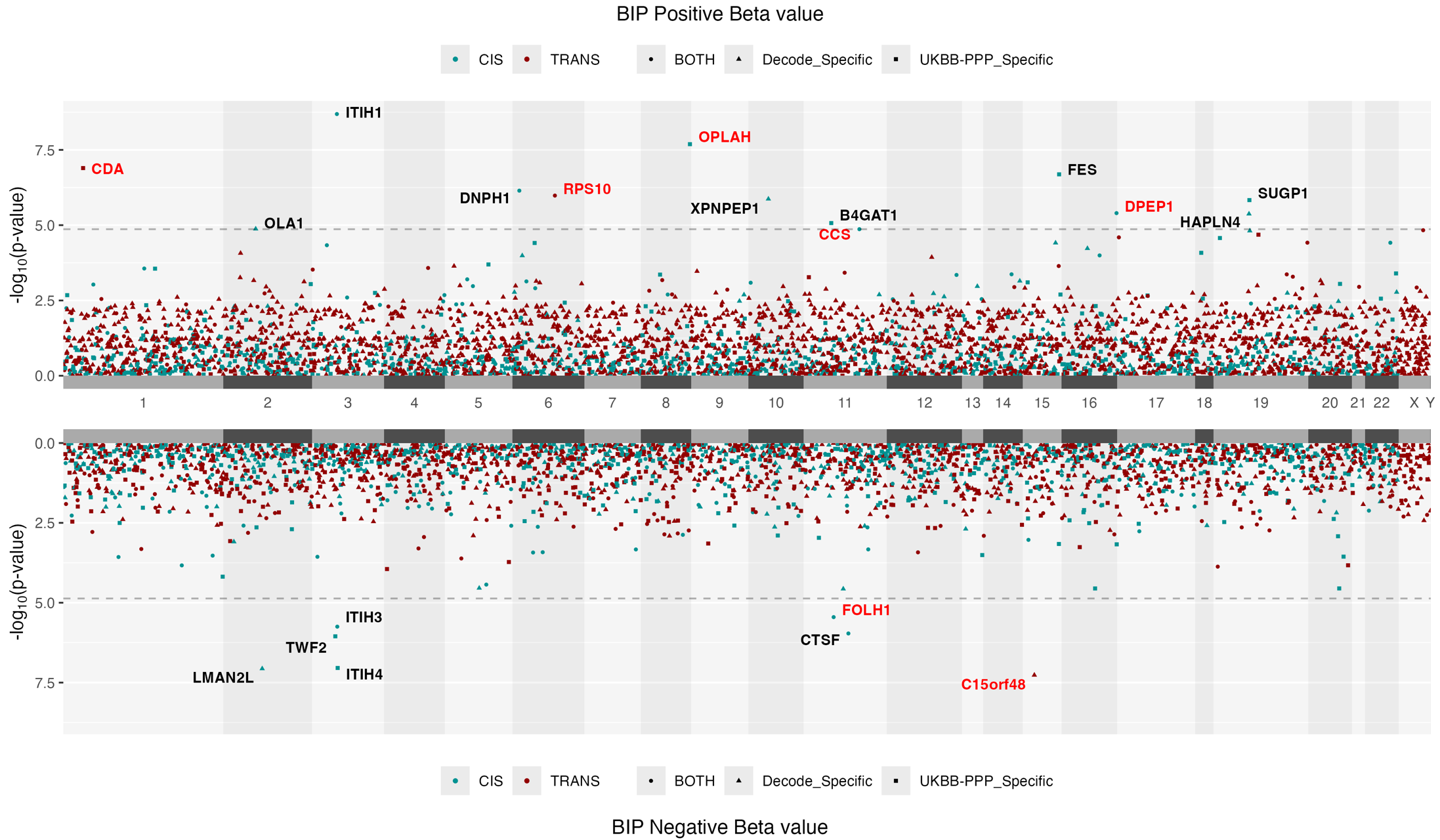
**

**2C. Major Depressive Disorder**

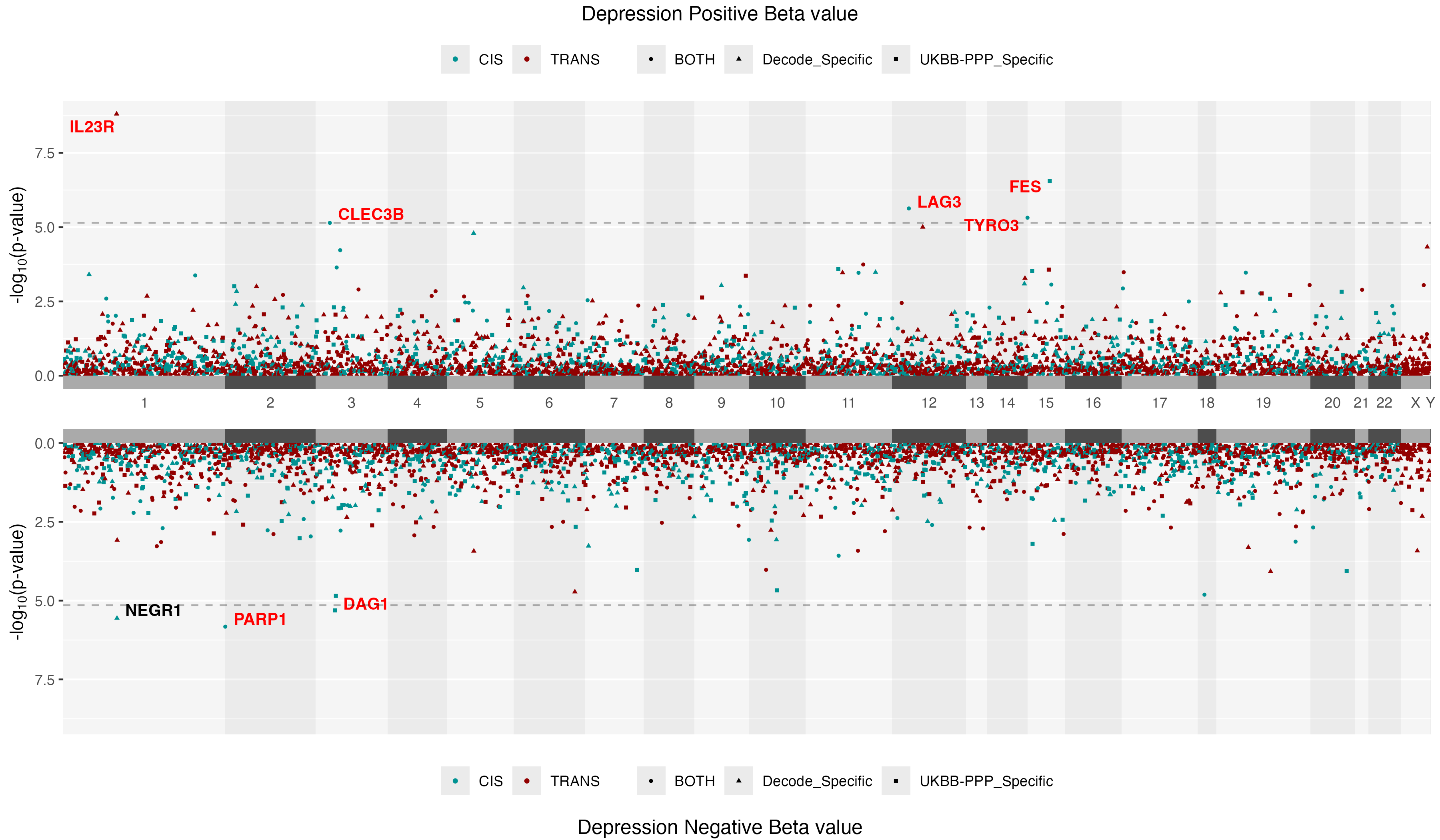


**2D. Cognitive Task Performance**


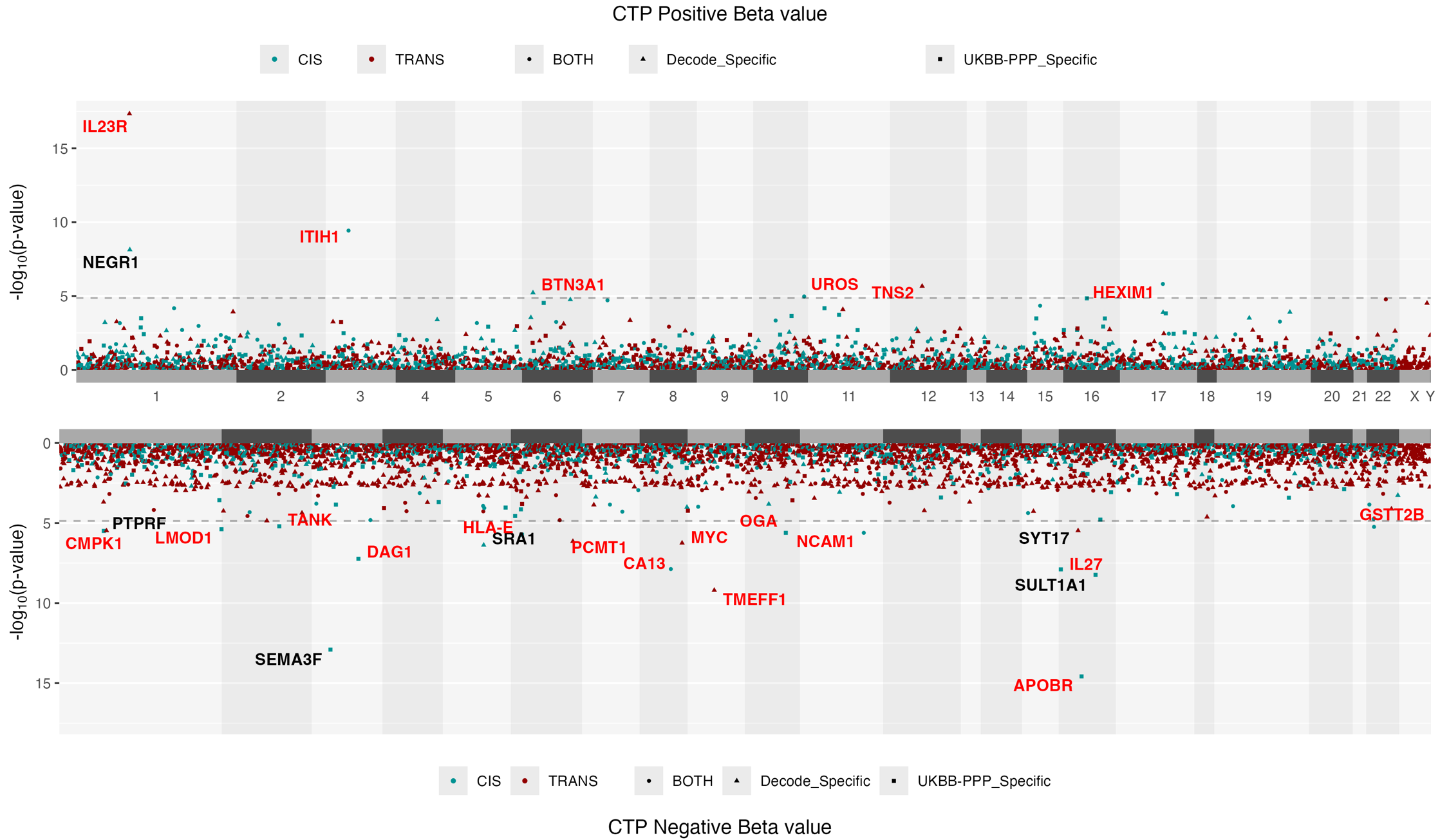


**eFigure 3A-Y: Forest plots, showing distribution of beta values across pQTLs for the 28 proteins that showed significant heterogeneity in eTable 2**

**3A.**

**3B.**

**3C.**

**3D.**

**3E.**

**3F.**

**3G.**

**MAPK3 BIP CIS-pQTL**

**3H.**

**3I.**

**3J.**

**3K.**

**3L.**

**3M.**

**3N.**

**3O.**

**3P.**

**3Q.**

**3R.**

**3S.**

**3T.**

**3U.**

**3V.**

**3W.**

**3X.**

**3Y.**

**eFigure 4. Correlation observed between Olink and SomaScan V4 platform using per- protein effect sizes (cis-pQTLs) obtained from MR analyses of A. schizophrenia, B.bipolar disorder, C. major depressive disorder, and D. cognitive task performance**

**4A. Schizophrenia**

**4B. Bipolar Disorder**

**4C. Major Depressive Disorder**

**4D. Cognitive Task Performance**

**eFigure 5: Correlation observed between Olink and SomaScan V4 platform using per- protein effect sizes (Trans-pQTLs without extended MHC) obtained from MR analyses of**

**A. schizophrenia, B. bipolar disorder, C. major depressive disorder, and D. cognitive task performance**

**5A. Schizophrenia**

**5B. Bipolar Disorder**

**5C. Major Depressive Disorder**

**.**

**5D. Cognitive Task Performance**

**eFigure 6: Protein-Protein interaction network obtained from StringDB for A. Schizophrenia, B. Bipolar Disorder, C. Major Depressive Disorder D. Cognitive task performance**

**6A. Schizophrenia**

**
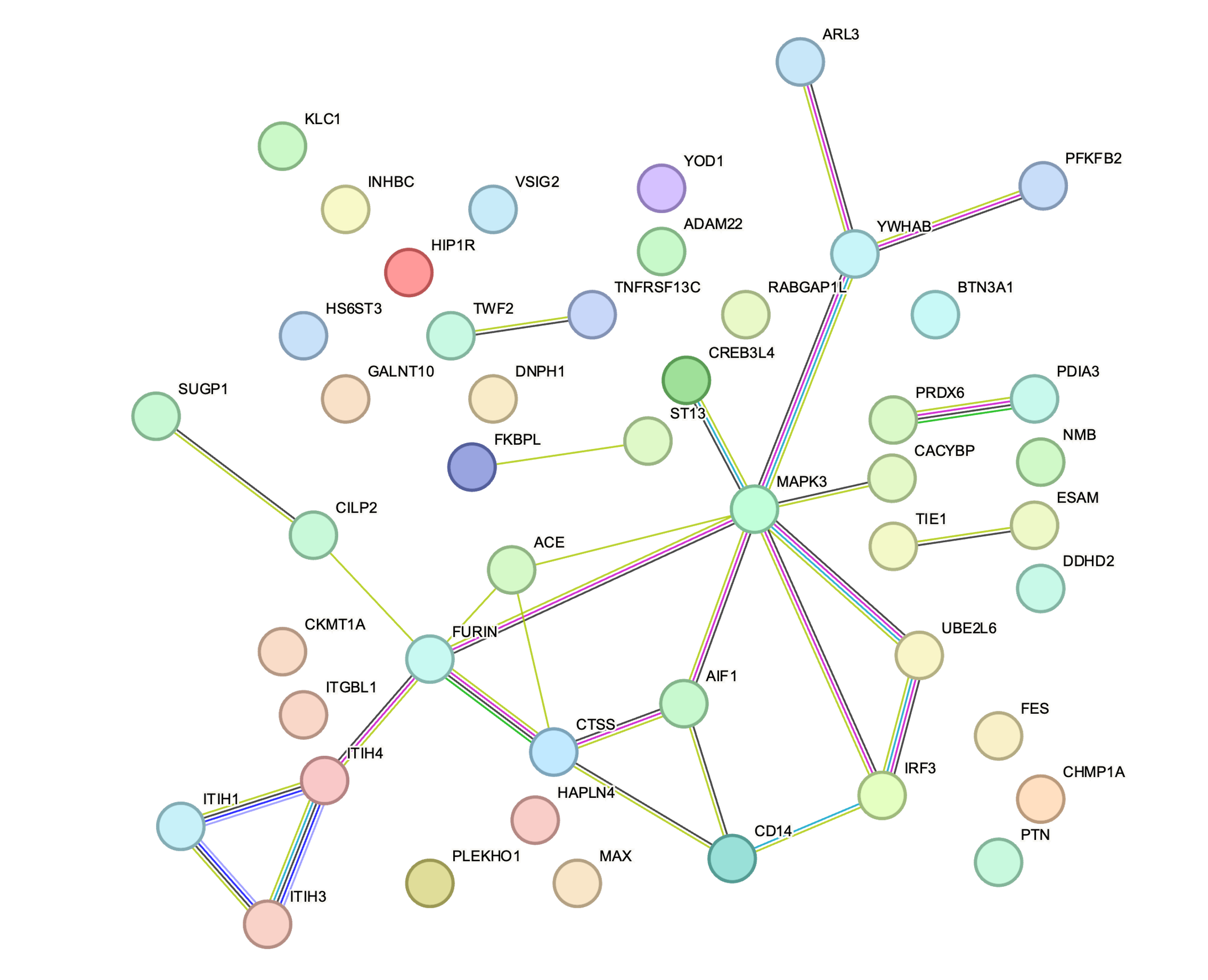
**

**3B. Bipolar Disorder**

**
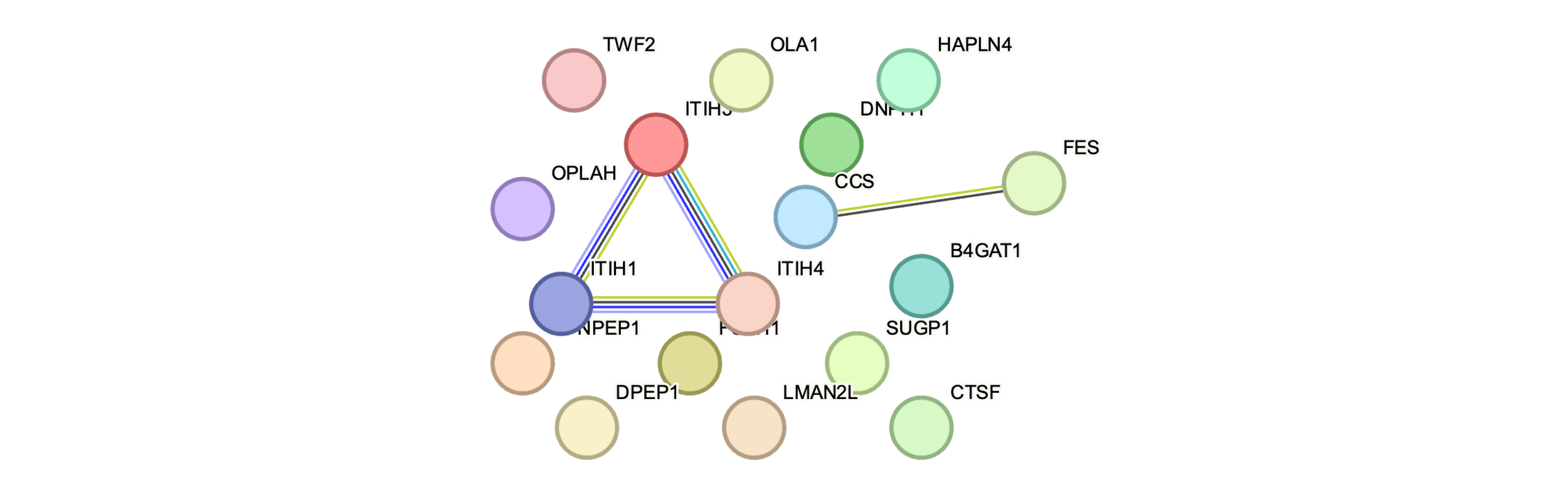
**

**3C. Major Depressive Disorder**

**
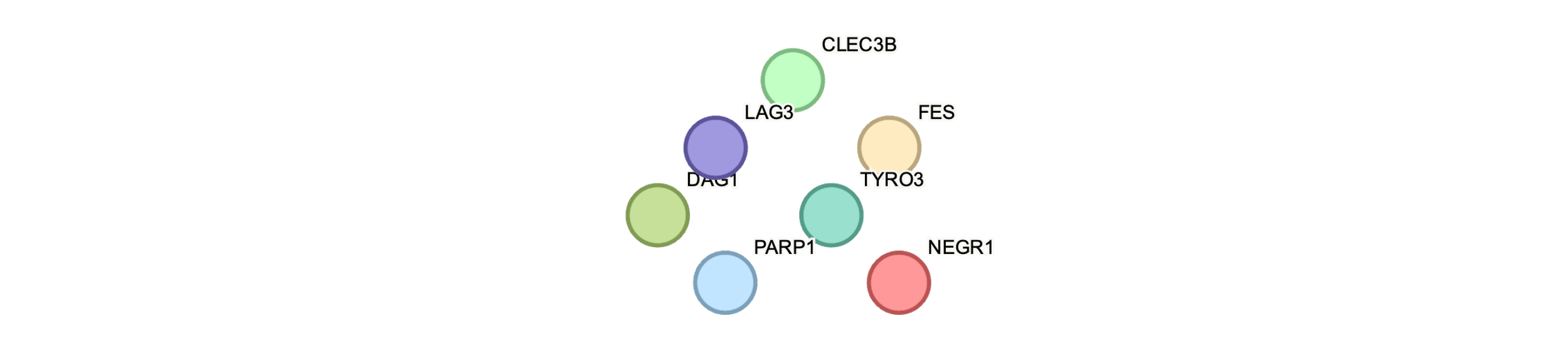
**

**3D. Cognitive Task Performance**

**
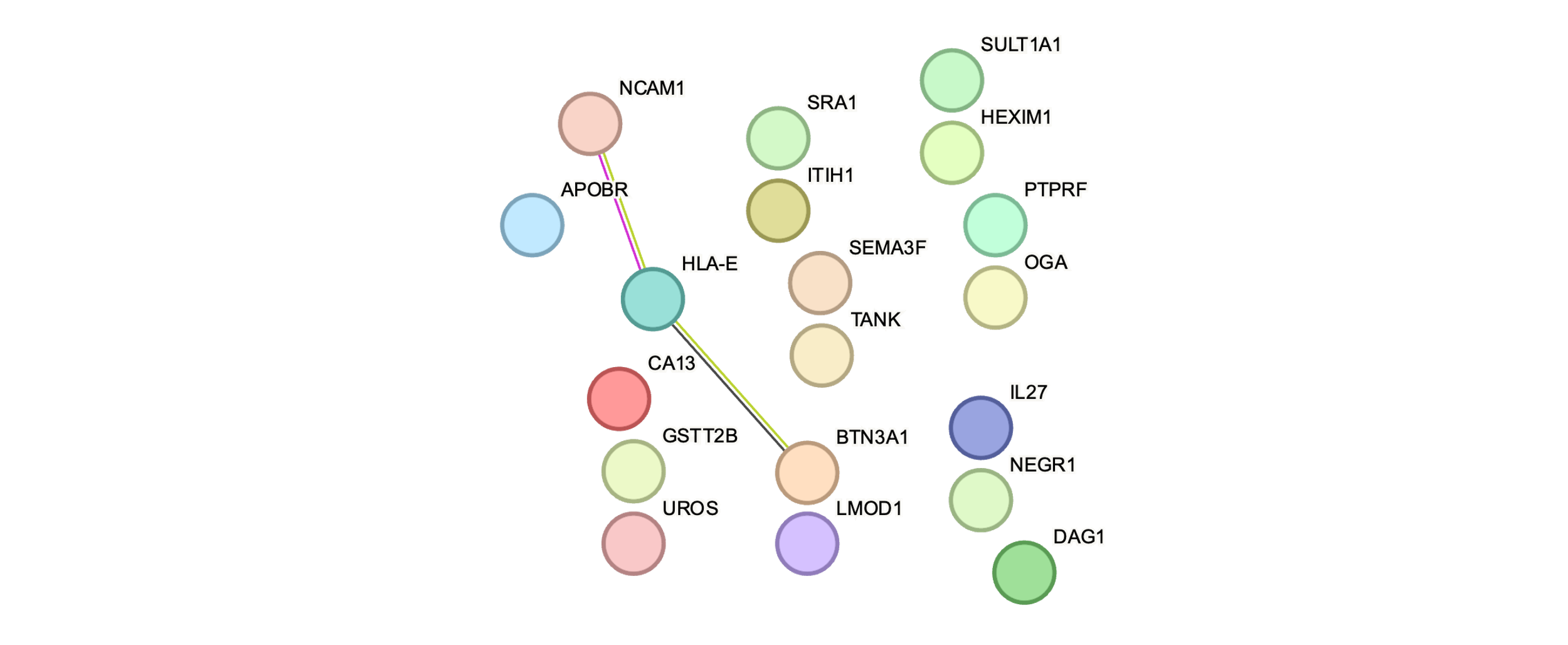
**
